# Diabetes Burden in the Middle East and North Africa Region, 1990–2023: An Ecological Time-Trend Analysis of GBD Estimates

**DOI:** 10.3390/medicina62071352

**Published:** 2026-07-13

**Authors:** Hanane Ouddoud, Judah Israel Ong Lescano, Keith Pardillada Belangoy, Yoshito Nishimura, Ko Harada, Hideharu Hagiya, Quynh Thi Vu, Naohiro Iwata, Tsukasa Higashionna, Tatsuaki Takeda, Yoshito Zamami, Toshihiro Koyama

**Affiliations:** 1 Department of Health Data Science, Graduate School of Medicine, Dentistry, and Pharmaceutical Sciences, Okayama University, Okayama 7008558, Japan; pes61rgo@s.okayama-u.ac.jp (H.O.);; 2 Department of Pharmacy, School of Health Care Professions, University of San Carlos, Cebu City 6000, Philippines; 3 Division of Hematology and Oncology, Mayo Clinic, Rochester, MN 55901, USA; 4 Brookdale Department of Geriatrics and Palliative Medicine, Icahn School of Medicine at Mount Sinai, New York, NY 10029, USA; 5 Department of Infectious Diseases, Okayama University Hospital, Okayama 7008558, Japan; 6 Faculty of Pharmacy, Haiphong University of Medicine and Pharmacy, Haiphong 180000, Vietnam; 7 Department of Pharmacy, Okayama University Hospital, Okayama 7008558, Japan; 8 Department of Education and Research Center for Clinical Pharmacy, Faculty of Pharmaceutical Sciences, Okayama University, Okayama 7008558, Japan

**Keywords:** GBD 2023, diabetes mellitus, Middle East and North Africa, joinpoint regression, cardiovascular risk factors, epidemiology, public health

## Abstract

*Background and Objectives*: The Middle East and North Africa (MENA) region has one of the highest age-standardized diabetes prevalence rates globally, yet all-age, diabetes-specific evidence incorporating GBD 2023 estimates through 2023 remains limited. *Materials and Methods*: Using Global Burden of Disease (GBD) 2023 estimates for 21 MENA countries from 1990 to 2023, this ecological time-trend analysis quantified 33-year trends in incidence, prevalence, mortality, and disability-adjusted life-years (DALYs); compared pre-2019 and post-2019 trajectories using joinpoint regression; characterized age- and sex-specific burden patterns; and quantified contributions of modifiable risk factors. *Results*: Age-standardized incidence increased 92%, from 251.7 (95% uncertainty interval [UI]: 231.5 to 272.4) to 482.5 (95% UI: 451.5 to 516.4) per 100,000, and prevalence more than doubled, from 5564 (95% UI: 5088 to 6024) to 11,247 (95% UI: 10,382 to 12,132) per 100,000. In 2023, males exhibited higher DALY rates than females in most adult age groups from age 15 years onward, shifting away from the female-predominant pattern seen in 1990; female rates remained higher at several of the oldest age groups. Children aged 0 to 14 years were the only group with declining DALY rates (−52% to −57%). Post-2019 incidence was higher in 15 of 21 countries, and six countries had higher DALY trends with non-overlapping confidence intervals. Because we only have four to five years of data, these short trends are preliminary and require care when evaluating. High body-mass index was the leading modifiable risk factor. *Conclusions*: These data support country-specific prevention and chronic-care strategies across the MENA region.

## 1. Introduction

Diabetes mellitus affected an estimated 589 million adults (aged 20–79 years) worldwide in 2024 and is projected to rise to 853 million by 2050 [1,2]; the Global Burden of Disease (GBD) study further projects that the all-age number of people living with diabetes could exceed 1.3 billion by 2050 [3]. Most of this burden is borne by low- and middle-income countries, where rapid urbanization and the nutrition transition continue to outpace public health responses [1,3].

In the Middle East and North Africa (MENA) region, estimates from both the GBD study and the International Diabetes Federation place the region among those with the highest age-standardized prevalence of diabetes [2,3]. This high burden has been attributed to rapid urbanization, high intake of energy-dense processed diets [4,5], obesity prevalence above 30% in several Gulf Cooperation Council (GCC) countries [6,7], male tobacco smoking rates exceeding 40% in multiple nations [8], and climate-related barriers to outdoor physical activity [9]. Nonetheless, the 21 MENA countries vary in income, infrastructure, and conflict exposure, spanning wealthy Gulf economies to fragile states; this heterogeneity contributes to substantial disparities in both disease burden and health system capacity [10,11].

Previous regional diabetes assessments have not yet fully incorporated all-age GBD 2023 estimates through 2023 or jointly examined post-2019 trends, sex-age distributions, and risk-attributable burden across all 21 GBD-defined MENA countries. Prior GBD-based studies of diabetes in MENA were limited to data through 2019 or 2021 [3,12] and therefore could not evaluate whether coronavirus disease 2019 (COVID-19), via direct beta-cell injury [13], lockdown-related behavioral shifts [9,14], or healthcare disruption [15,16], has altered population-level diabetes trajectories. Another gap concerns sex-specific burden: although a global shift toward male predominance in diabetes has been reported [3,17], it remains unclear whether this trend applies to MENA. In the region, obesity is more common in women, whereas smoking is more common in men. How these different risks affect the overall gap in diabetes burden between the sexes is still not well understood across MENA populations.

To extend previous studies [3,12], we incorporate GBD 2023 estimates across all 21 MENA countries. We examine both absolute counts and age-standardized rates to illustrate the influence of population growth and aging in the region. We also analyze sex- and age-specific patterns, as well as risk-attributable burden, and group countries by their burden profiles to provide specific, realistic policy recommendations.

The objectives of this study were to quantify 33-year trends in incidence, prevalence, mortality, and disability-adjusted life-years (DALYs); evaluate the descriptive role of demographic changes; compare pre- and post-2019 trajectories using exploratory joinpoint regression; and analyze the contributions of modifiable risk factors.

## 2. Materials and Methods

### 2.1. Study Design and Data Source

This ecological time-trend analysis was based on publicly available estimates from the GBD 2023 study, which provides comprehensive and comparable estimates for 375 diseases and injuries across 204 countries and territories from 1990 to 2023. The GBD study is coordinated by the Institute for Health Metrics and Evaluation (IHME) at the University of Washington. Diabetes mellitus burden was assessed across 21 MENA countries from 1990 to 2023: Afghanistan, Algeria, Bahrain, Egypt, Iran, Iraq, Jordan, Kuwait, Lebanon, Libya, Morocco, Oman, Palestine, Qatar, Saudi Arabia, Sudan, Syria, Tunisia, Türkiye, United Arab Emirates (UAE), and Yemen. The study adhered to the Guidelines for Accurate and Transparent Health Estimates Reporting (GATHER) statement [18] and the Strengthening the Reporting of Observational Studies in Epidemiology (STROBE) guidelines [19].

Data were extracted from the GBD Results Tool (https://vizhub.healthdata.org/gbd-results/; accessed 10 January 2026). For this analysis, we extracted absolute counts and age-standardized rates of diabetes incidence, prevalence, mortality, years of life lost (YLLs), years lived with disability (YLDs), and DALYs across all age groups, for both sexes combined and separate sexes from 1990 to 2023. We also collected data for behavioral, environmental, and metabolic risk factors. Because primary records are limited in conflict areas (Afghanistan, Libya, Palestine, Sudan, Syria, and Yemen), these GBD data represent statistical estimates rather than direct registry counts.

### 2.2. Case Definition

We studied diabetes mellitus as a Level 3 cause within the GBD hierarchy, defined by the International Statistical Classification of Diseases, 10th Revision (ICD-10) codes E10–E14 [20]. Although GBD provides estimates for type 1 and type 2 diabetes at Level 4, primary data in many MENA countries do not consistently distinguish between these subtypes. To keep the data consistent across all 21 countries, we used the combined Level 3 ‘Diabetes mellitus’ category [21].

### 2.3. Epidemiological Metrics

Incidence, prevalence, mortality, YLLs, YLDs, and DALYs were extracted. Age-standardized rates (ASRs) per 100,000 population were calculated by direct standardization to the GBD 2023 global reference population. All metrics are reported with 95% uncertainty intervals (UIs), obtained from the GBD 2023 outputs as the 2.5th and 97.5th percentiles of the posterior distribution [21].

### 2.4. Risk Factor Assessment

We studied diabetes burden from high body-mass index (BMI), low physical activity, smoking, secondhand smoke, particulate matter pollution (PM), and dietary risks (including diets high in sugar-sweetened beverages, red meat, and processed meat, and diets low in whole grains, fruits, vegetables, and fiber) using GBD methods [21]. High fasting plasma glucose was not included because it acts as a metabolic mediator of diabetes. Population attributable fractions (PAFs) were computed relative to the theoretical minimum-risk exposure level (TMREL) for each risk factor and adjusted for mediation in the GBD framework to prevent double-counting; thus, attributable burdens for different risk factors are not mutually exclusive and cannot be added together. These risk-attributable DALY rates and their 95% UIs are reported in Table S8.

### 2.5. Estimation Methods

Within the GBD 2023 framework, IHME estimated diabetes incidence and prevalence using DisMod-MR 2.1, a Bayesian meta-regression tool synthesizing population surveys, hospital records, disease registries, and published literature while enforcing epidemiological consistency [22]. To address data sparsity and heterogeneity, spatiotemporal Gaussian process regression (ST-GPR) was employed. This framework utilizes country-level covariates to pool information across locations and time, allowing for robust estimation in data-limited settings. Mortality estimates were generated using the Cause of Death Ensemble model (CODEm), which integrates multiple predictive models and adjusts for non-specific coding through the redistribution of garbage codes [21].

### 2.6. Statistical Analysis

Temporal trends in age-standardized rates (incidence, prevalence, mortality, and DALYs) from 1990 to 2023 were analyzed using the Joinpoint Regression Program (version 6.0.1; National Cancer Institute, Bethesda, MD, USA). Standard errors for each annual rate were derived from the GBD 95% uncertainty intervals as SE = (upper − lower)/3.92 and supplied to the regression using the program’s standard-error (heteroscedastic) variance option, which weights each observation by the inverse of its variance, so that the model accounted for the precision of each annual estimate. Rates were log-transformed so that segment slopes represent the annual percent change (APC). Joinpoints were located by a grid search with a minimum of two observations before the first joinpoint, after the last joinpoint, and between joinpoints, and a maximum of five joinpoints. The optimal number of joinpoints was selected by the permutation test (4499 permutations; overall α = 0.05) [23]. For each segment, the APC and its 95% confidence interval (CI) were estimated, and the average annual percent change (AAPC) was calculated for 1990–2023, 1990–2019, and 2019–2023 using the empirical quantile method (5001 resamples). A trend was considered statistically significant when its 95% CI excluded zero.

We report 95% UIs for GBD estimates and 95% CIs for joinpoint results. Because the joinpoint regression incorporated the GBD uncertainty intervals as standard errors, the reported CIs reflect both sampling variation and GBD estimation uncertainty. The short post-2019 segment (four to five data points) remains statistically fragile; to assess post-2019 changes, we compared pre- and post-2019 AAPCs using non-overlapping CIs rather than a formal statistical test, and because we did not adjust for multiple comparisons these post-2019 trends are exploratory. Data processing and visualization were performed using R (version 4.2.2; R Foundation for Statistical Computing, Vienna, Austria) and Microsoft Excel (Microsoft Corporation, Redmond, WA, USA).

## 3. Results

### 3.1. Regional Temporal Trends

Between 1990 and 2023, diabetes burden increased across the MENA region by all metrics (Tables S1–S5). In absolute terms, incident cases increased by 394.9%, from 0.64 million (95% UI: 0.59 to 0.69) in 1990 to 3.19 million (95% UI: 2.96 to 3.41) in 2023. Prevalent cases increased by 468.1%, from 11.7 million (95% UI: 10.7 to 12.8) to 66.6 million (95% UI: 61.3 to 72.1). Diabetes-related deaths increased by 234.0%, from 38.1 thousand (95% UI: 25.4 to 53.4) to 127.2 thousand (95% UI: 104.5 to 152.4), and total DALYs increased by 322.6%, from 1.90 million (95% UI: 1.45 to 2.36) to 8.02 million (95% UI: 6.28 to 9.80).

**Figure 1:**
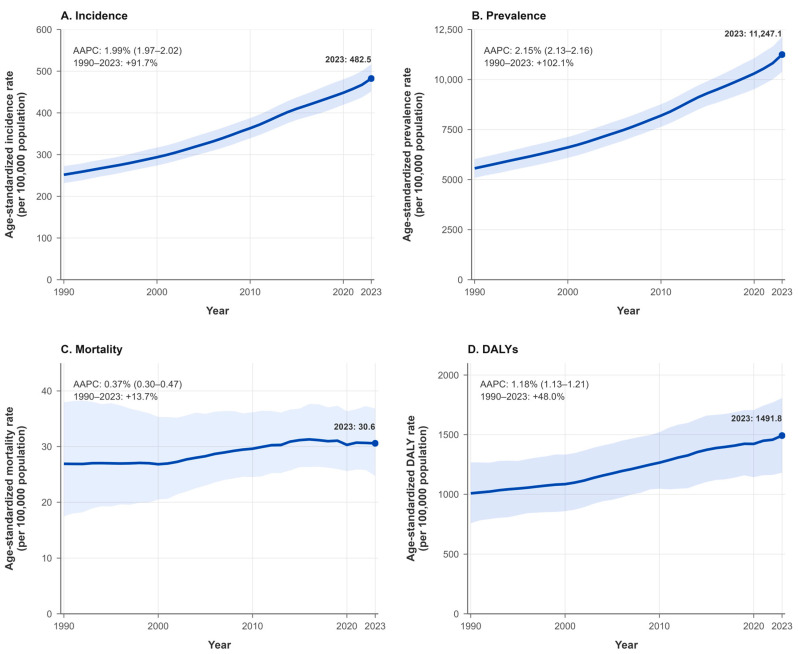
Temporal trends in age-standardized diabetes burden across the MENA region, 1990–2023. (**A**) Incidence rate per 100,000. (**B**) Prevalence rate per 100,000. (**C**) Mortality rate per 100,000. (**D**) Disability-adjusted life-year (DALY) rate per 100,000. Solid lines represent point estimates from the Global Burden of Disease (GBD) 2023 study; shaded areas represent 95% uncertainty intervals. Annotations indicate the average annual percent change (AAPC) for the full period (1990–2023) with 95% confidence intervals. Solid circles mark 2023 endpoint values. AAPC, average annual percent change; CI, confidence interval; DALY, disability-adjusted life-year; GBD, Global Burden of Disease; MENA, Middle East and North Africa; UI, uncertainty interval.

After age standardization, these increases were attenuated but remained substantial. Incidence rose by 92%, from 251.7 (95% UI: 231.5 to 272.4) to 482.5 (95% UI: 451.5 to 516.4) per 100,000 (AAPC: 1.99%; 95% CI: 1.97 to 2.02; *p* < 0.001; Figure 1A). Prevalence more than doubled, from 5564 (95% UI: 5088 to 6024) to 11,247 (95% UI: 10,382 to 12,132) per 100,000 (AAPC: 2.15%; 95% CI: 2.13 to 2.16; *p* < 0.001; Figure 1B). Mortality increased from 26.9 (95% UI: 17.5 to 38.0) to 30.6 (95% UI: 24.7 to 36.8) per 100,000 (AAPC: 0.37%; 95% CI: 0.30 to 0.47; *p* < 0.001; Figure 1C). The DALY rate increased by 48%, from 1008 (95% UI: 760 to 1268) to 1492 (95% UI: 1181 to 1807) per 100,000 (AAPC: 1.18%; 95% CI: 1.13 to 1.21; *p* < 0.001; Figure 1D).

### 3.2. Sex- and Age-Specific Patterns

**Figure 2:**
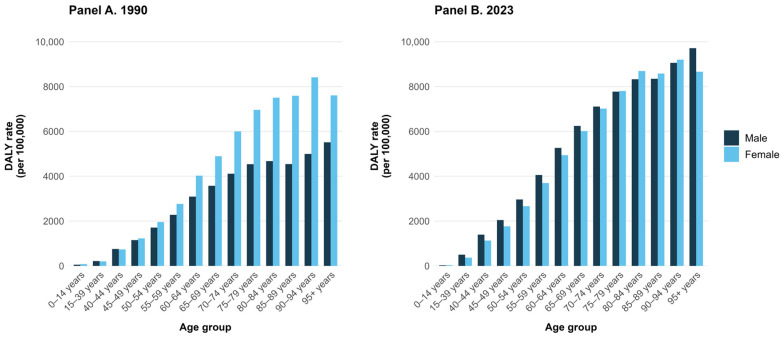
Age- and sex-specific diabetes DALY rates in the MENA region, 1990 versus 2023. (**A**) DALY rates per 100,000 by age group and sex, 1990. (**B**) DALY rates per 100,000 by age group and sex, 2023. Dark blue bars represent males; light blue bars represent females. Data are pooled estimates for the entire MENA region from the Global Burden of Disease (GBD) 2023 study. DALY, disability-adjusted life-year; MENA, Middle East and North Africa.

In 1990, DALY rates were higher in females than in males across most adult age groups, particularly from ages 45 to 95+ years, with the widest gap in the 50 to 79-year range. By 2023, males had higher DALY rates than females in most age groups from 15 years onward, although female rates remained higher at several of the oldest age groups (Figure 2). The magnitude of change differed by sex. Across ages 55 to 95+, male DALY rates rose by 70 to 84%, whereas female rates increased by 9 to 34%. The male-to-female ratio shifted most at ages 75 to 79, increasing from 0.65 in 1990 to 0.89 in 2023 (Figure S1). Among younger adults (15 to 39 years), male DALYs increased by 133% (from 216 to 502 per 100,000), compared with an 83% increase in females (from 202 to 369 per 100,000). Children aged 0 to 14 years were the only group with declining DALYs: −57% in females (from 78.9 to 33.7 per 100,000) and −52% in males (from 55.1 to 26.3 per 100,000).

### 3.3. Country-Level Heterogeneity and Post-2019 Trends

The 21 MENA countries showed substantial between-country variation (Table 1; Tables S1–S4). In 2023, DALY rates varied by 3.6-fold, ranging from 973.7 (95% UI: 727.5 to 1255.4) per 100,000 in Yemen to 3460.6 (95% UI: 2807.8 to 4214.6) per 100,000 in Bahrain. Saudi Arabia had the highest incidence (899.4; 95% UI: 839.2 to 963.5 per 100,000), whereas Lebanon had the lowest mortality rate (10.90; 95% UI: 8.43 to 14.12 per 100,000).

**Figure 3:**
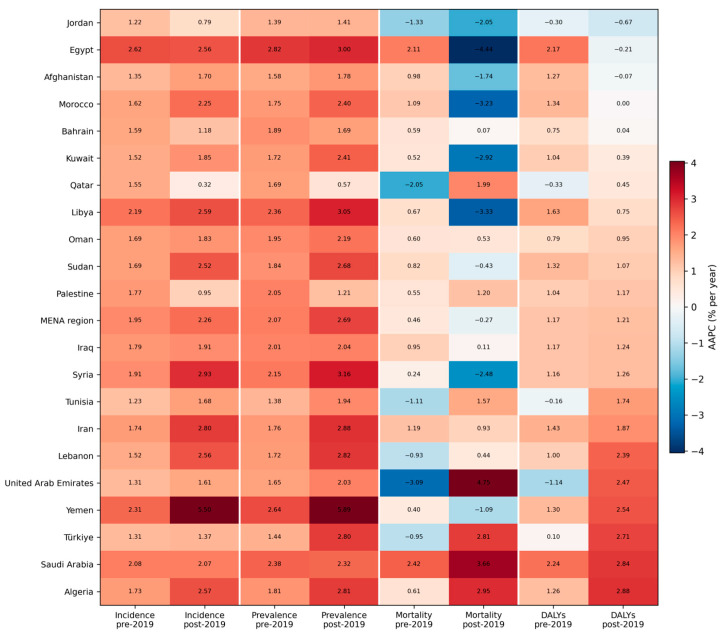
Comparison of pre-2019 (1990–2019) and post-2019 (2019–2023) average annual percent changes (AAPCs) in age-standardized diabetes burden by country and metric. The heatmap displays the estimated AAPC (% per year) for each of the 21 Middle East and North Africa countries across four GBD metrics (incidence, prevalence, mortality, and DALYs), stratified by pre- and post-2019 periods. Color intensity represents AAPC magnitude: red shading indicates higher rates of increase; blue shading indicates lower rates of increase or decline. AAPC, average annual percent change; DALY, disability-adjusted life-year.

**Figure 4:**
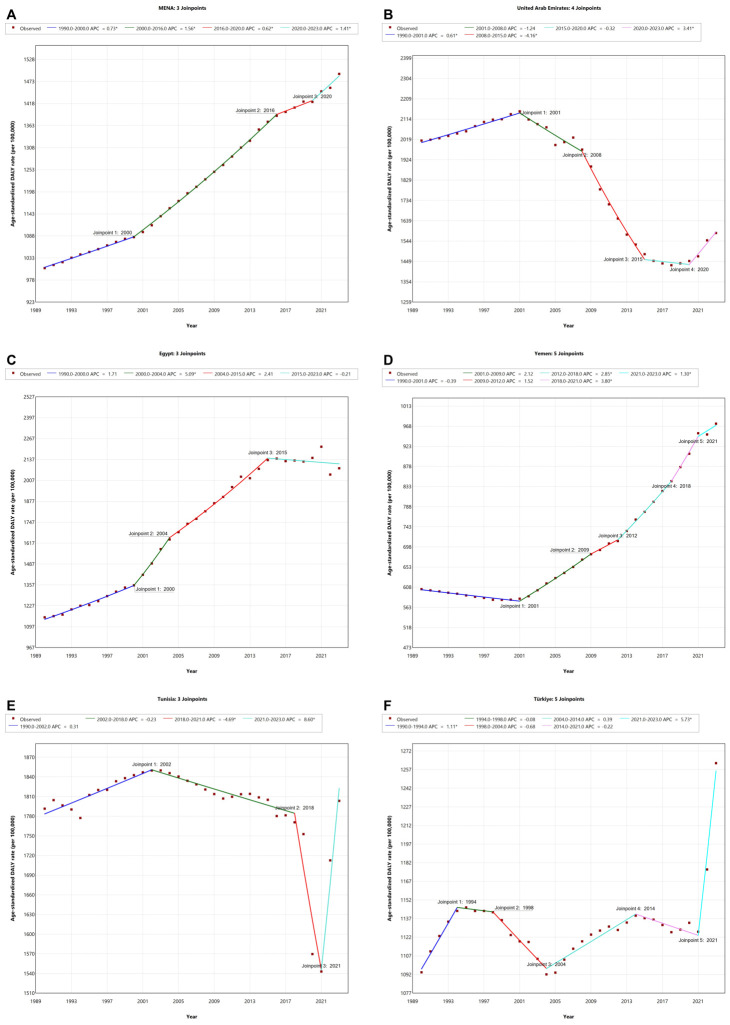
Joinpoint-fitted trends in age-standardized diabetes disability-adjusted life-year (DALY) rates, MENA region and selected countries, 1990–2023. (**A**) MENA region; (**B**) United Arab Emirates; (**C**) Egypt; (**D**) Yemen; (**E**) Tunisia; (**F**) Türkiye. Points represent observed Global Burden of Disease (GBD) 2023 estimates; lines represent fitted joinpoint segments, with annotations indicating segment-specific annual percent changes (APCs) and identified joinpoints (years at which the trend changed). Asterisks denote APCs significantly different from zero (*p* < 0.05). Trajectories for the remaining MENA countries are shown in Figure S2. APC, annual percent change; DALY, disability-adjusted life-year; GBD, Global Burden of Disease; MENA, Middle East and North Africa.

Pre- and post-2019 AAPC confidence intervals for DALY trends did not overlap in six countries (Figure S3; Table S7), while five other countries (Afghanistan, Bahrain, Egypt, Libya, and Morocco) had lower post-2019 trends with non-overlapping intervals. These higher post-2019 trends were observed in: Türkiye (pre-2019 AAPC: 0.10%, 95% CI: 0.05 to 0.15; post-2019 AAPC: 2.71%, 95% CI: 2.29 to 3.08), UAE (pre-2019: −1.14%, 95% CI: −1.28 to −0.97; post-2019: 2.47%, 95% CI: 1.27 to 3.51), Tunisia (pre-2019: −0.16%, 95% CI: −0.32 to 0.09; post-2019: 1.74%, 95% CI: 0.24 to 3.13), Yemen (pre-2019: 1.30%, 95% CI: 1.26 to 1.37; post-2019: 2.54%, 95% CI: 2.10 to 2.85), Lebanon (pre-2019: 1.00%, 95% CI: 0.92 to 1.08; post-2019: 2.39%, 95% CI: 1.84 to 3.19), and Algeria (pre-2019: 1.26%, 95% CI: 1.20 to 1.37; post-2019: 2.88%, 95% CI: 1.98 to 3.44) (Figure 3 and Figure 4).

### 3.4. Risk Factor Attribution

**Figure 5:**
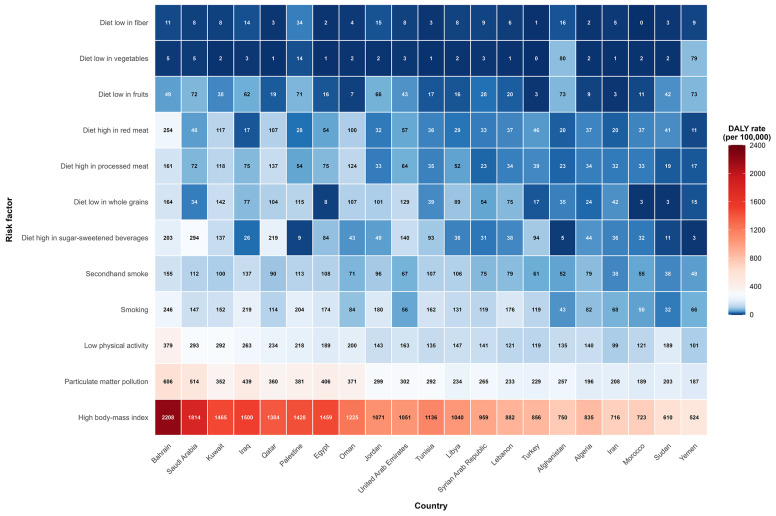
Risk factor-attributable diabetes DALY rates across 21 MENA countries, 2023. Heatmap showing age-standardized DALY rates per 100,000 population attributable to modifiable risk factors for diabetes in 2023, for both sexes combined. Cell values represent attributable DALY rates. Warmer colors (red) indicate greater attributable burden, whereas cooler colors (blue) indicate lower attributable burden. Countries and risk factors are arranged from higher to lower mean attributable burden. High fasting plasma glucose is excluded as a metabolic mediator. BMI, body-mass index; DALY, disability-adjusted life-year; MENA, Middle East and North Africa; PM, particulate matter pollution.

High BMI was the leading modifiable risk factor for diabetes DALYs across all 21 countries in 2023. BMI-attributable DALY rates ranged from 524 per 100,000 in Yemen to 2208 per 100,000 in Bahrain (Figure 5; Table S8). After high BMI, PM was the second-largest contributor in all 21 countries, contributing 187 to 606 DALYs per 100,000. Tobacco smoking-attributable DALYs exceeded 150 per 100,000 in eight countries (Bahrain, Iraq, Palestine, Jordan, Lebanon, Egypt, Tunisia, and Kuwait); dietary risks were the next-largest contributor in most countries.

### 3.5. Disability Composition

The YLD fraction of diabetes DALYs ranged from 42.5% in Bahrain to 81.8% in Lebanon (Table S9). The highest YLD fractions were observed in Lebanon, Libya, Syria, UAE, and Kuwait, whereas the highest YLL fractions were observed in Bahrain, Oman, Tunisia, Iraq, and Palestine.

## 4. Discussion

GBD 2023 data show increases in diabetes burden across MENA countries from 1990 to 2023. All the age-standardized incidence, prevalence, and DALY rates increased, and the rise in absolute counts was even larger. This points to population growth and aging as contributors to demand for diabetes care across the region. The burden also shifted toward male predominance across most adult age groups, and post-2019 incidence trends were higher in most countries.

### 4.1. Scale of the Burden

MENA’s diabetes DALY rate reached 1492 per 100,000 in 2023, placing it among the highest-burden regions globally [3,21]. In the MENA region, absolute increases in diabetes incidence, prevalence, deaths, and DALYs far exceeded age-standardized trends. For example, absolute DALYs increased by 322.6% compared to a 48% increase in the age-standardized DALY rate. This difference is consistent with population growth and aging contributing to the rising absolute demand on health systems [3,21].

Age-standardized prevalence more than doubled between 1990 and 2023, while DALY rates increased by 48%. These trends indicate that more people are living longer with diabetes, which increases the cumulative burden of disability and the need for long-term care. This aligns with global and regional estimates, where type 2 diabetes prevalence has increased faster than age-standardized DALY and mortality rates [3,24]. Lebanon is a notable example, reporting the region’s lowest age-standardized mortality in 2023 (10.9 per 100,000) but the highest YLD fraction, with 81.8% of DALYs driven by disability.

### 4.2. The Shift from Female to Male Predominance

Between 1990 and 2023, the MENA region experienced a shift from female to male predominance in DALY burden across most adult age groups. Among adults aged 55 years and older, female DALY rates increased by 9 to 34%, compared with 70 to 84% in males. In young adults (15 to 39 years), male DALY rates increased by 133% compared with 83% in females.

These patterns reflect sex-specific risk profiles. In Saudi Arabia, obesity is more prevalent in women, whereas smoking and dyslipidemia are more common in men [25]. Data from Jordan, Lebanon, Syria, and Palestine show higher tobacco use in men [8,26,27]. While physical inactivity remains high among GCC women due to social and environmental barriers [28], the 133% increase in male DALY rates at ages 15 to 39 years points to rapid risk accumulation among younger men in less active urban settings, compounded by tobacco exposure and rising adiposity. Children (aged 0 to 14 years) were the only cohort with declining DALY rates in both sexes. Because diabetes in children is mainly type 1 (T1DM), this decline may partly reflect clinical improvements, such as earlier diagnosis and better access to insulin, rather than changes in metabolic risk factors [29].

### 4.3. Post-2019 Trends

Post-2019 incidence trends were higher in 15 of 21 MENA countries compared to the pre-2019 period (Figure S3). This rise was in the same years as the COVID-19 pandemic and regional risk factors, including obesity and physical inactivity [9,10,11,14]. These trends are preliminary and require further research. While clinical studies have reported associations between COVID-19 infection and new-onset diabetes [30,31,32], the population-level contribution of these biological mechanisms in MENA cannot be determined from ecological data alone. Health-service disruption could have also contributed through delayed diagnosis, interrupted follow-up, reduced access to routine care, and rebound detection after services resumed [15,16]. Reports from the Eastern Mediterranean Region show that the pandemic disrupted essential non-communicable disease services and continuity of chronic care, including medication access and follow-up [16,33,34,35]. These factors may explain the higher post-2019 DALY trends (non-overlapping pre- and post-2019 CIs) observed in six countries: Türkiye, UAE, Tunisia, Yemen, Lebanon, and Algeria.

While some countries followed their long-term trajectories, Egypt showed a reversal. Jordan, Qatar, and Bahrain experienced continued incidence increases despite stable or declining mortality [10,11,15,16]. Yemen showed the largest post-2019 increase in incidence (5.50%; 95% CI: 5.37 to 5.73). Because of the conflict in Yemen, this rise could be due to several factors, such as rising obesity, interrupted health tracking, or population displacement [36,37,38]. Similar challenges exist in Sudan and other fragile states, where post-2019 shifts reflect both epidemiological changes and reduced visibility of chronic disease during conflict.

### 4.4. Country-Level Variation and Risk Factors

Diabetes burden varied across the 21 MENA countries. The 3.6-fold gap in DALY rates between Yemen and Bahrain may reflect major differences in urbanization, diet, obesity, tobacco exposure, health-system capacity, and conflict-related disruption [5,6,7,8,9,10,11,35,36,37]. World Bank data show a large range in urbanization across the region, from about 26% in Afghanistan to over 99% in Kuwait [39]. Bahrain illustrates this: it has among the highest diabetes DALY and mortality rates in the region. Recent primary-care data from Bahrain confirm that over half of patients had at least one diabetes-related complication [5,6,7,9,40].

### 4.5. Implications for Health Systems

Rising absolute case counts require health systems across MENA to plan for increased demand in diagnosis, glucose monitoring, complication screening, and long-term treatment. Second, countries with higher post-2019 trends need stronger surveillance to determine if recent increases represent a real epidemiological shift or delayed detection. Third, the shift toward male predominance in young adults aged 15 to 39 years shows the need for screening and prevention programs for younger males.

Focusing on obesity alone is insufficient. High BMI is the leading risk factor, but dietary risks, smoking, and PM pollution are also major contributors. The biological link between PM and diabetes is not as clear as the obesity link, but data show a connection via systemic inflammation [41]. Since many MENA countries have PM levels above WHO guidelines, air pollution is a relevant factor for regional policy. In several countries, air pollution-attributable DALY rates were higher than tobacco rates.

Policy responses should address local needs. Countries with high YLD burdens, such as Lebanon, Libya, Syria, UAE and Kuwait, require chronic-care models that focus on retinal and renal screening, foot care, and self-management support. Mortality-dominant countries with high YLL burdens, such as Bahrain, Oman, Tunisia, Iraq and Palestine need early diagnosis to prevent death. Countries with high obesity in the Gulf need sugar-sweetened beverage taxes and healthy food policies, which work in other countries [42,43]. For example, the sugary drink taxes in Saudi Arabia and the UAE in 2017 helped reduce sales and sugar intake [44]. Countries with post-2019 DALY trend increases (Türkiye, UAE, Tunisia, Yemen, Lebanon, and Algeria) need better health tracking to check if these increases are real or from delayed diagnosis. In conflict countries (Afghanistan, Libya, Palestine, Sudan, Syria, and Yemen), recommendations must be practical because of the weak health tracking and limited healthcare access. The priority is to provide insulin and essential diagnostics [37,38].

### 4.6. Strengths and Limitations

Strengths of this study include its 33-year period, using standard GBD methods to compare 21 countries. We also combined several analyses, such as YLD/YLL decomposition, sex-stratified trends, risk factors, and pre- and post-2019 comparisons.

GBD estimates are model-based and use regional primary data of different quality. In conflict countries (Afghanistan, Libya, Palestine, Sudan, Syria, and Yemen), population movement and incomplete local registries affect the estimates. Also, because this is an ecological study, we cannot study individual cases or local differences. The post-2019 analyses are also statistically fragile. Although our joinpoint models incorporated the GBD uncertainty intervals as standard errors, these intervals may not fully capture all sources of error inherent in model-based estimates. The short post-2019 period (four to five data points) and the lack of multiple comparisons mean these findings are preliminary. Also, because we used the combined diabetes category, we did not separate type 1 from type 2 diabetes in our analysis. This makes our results for children and risk factors uncertain. Finally, the relative risks we used for risk factors come from international studies and might not represent MENA populations. Also, because we did not perform a mathematical decomposition, our discussion of population growth and aging remains descriptive.

## 5. Conclusions

Diabetes burden in MENA increased between 1990 and 2023 across incidence, prevalence, mortality, and DALYs, with a larger rise in absolute counts than in ASRs, pointing to a contribution from population growth and aging. The burden also shifted toward male predominance across most adult age groups, with the largest increases in younger men.

The wide variation across the 21 countries shows that a single regional strategy will not be sufficient. Gulf countries share a rapid nutrition transition, high obesity burden, and climate-related barriers to outdoor activity, and need stronger structural policies targeting obesity and tobacco use. Countries with disability-dominant burden need sustained investment in chronic care. Conflict-affected settings need resilient systems that can maintain continuity of care during disruption.

## Figures and Tables

**Table 1:** Summary of age-standardized regional and selected country-level diabetes burden, 2023. Age-standardized incidence, prevalence, mortality, and disability-adjusted life-year (DALY) rates per 100,000 population for diabetes mellitus in selected countries of the Middle East and North Africa (MENA) region in 2023, based on GBD 2023 estimates. Values are presented as rate (95% uncertainty interval) for both sexes combined. The regional aggregate is shown for comparison. The full 21-country dataset is reported in Table S6. DALY, disability-adjusted life-year; UI, uncertainty interval.

Country	Incidence Rate * [95% UI]	Prevalence Rate * [95% UI]	Mortality Rate * [95% UI]	DALY Rate * [95% UI]
Middle East and North Africa	482.5 [451.5–516.4]	11,247.1 [10,381.5–12,131.8]	30.6 [24.7–36.8]	1491.8 [1180.9–1807.0]
Bahrain	770.2 [710.1–823.4]	18,377.7 [16,881.7–19,690.8]	117.0 [88.4–146.9]	3460.6 [2807.8–4214.6]
Egypt	590.8 [551.1–633.8]	13,952.8 [12,943.8–15,091.4]	48.4 [36.3–63.3]	2082.4 [1660.0–2505.5]
Lebanon	499.0 [460.3–538.2]	12,323.3 [11,305.6–13,324.8]	10.9 [8.4–14.1]	1277.5 [949.3–1653.5]
Saudi Arabia	899.4 [839.2–963.5]	23,138.3 [21,309.0–25,001.5]	58.7 [43.5–74.3]	2648.6 [2112.6–3280.2]
Tunisia	449.4 [416.3–486.0]	10,362.4 [9520.4–11,264.9]	52.1 [41.6–65.9]	1803.5 [1448.6–2179.3]
Türkiye	374.9 [344.5–409.4]	8599.5 [7815.9–9433.6]	27.8 [21.1–34.9]	1262.2 [968.7–1551.2]
Yemen	307.5 [277.5–338.1]	7298.8 [6509.6–7995.4]	19.3 [11.9–29.4]	973.7 [727.5–1255.4]

* Rates are age-standardized per 100,000 population.

## Data Availability

All source data are publicly accessible through the GBD Results Tool (https://vizhub.healthdata.org/gbd-results/ (accessed on 10 January 2026)). Processed data, analysis code, and figure-generation scripts have been deposited at Zenodo (doi: 10.5281/zenodo.19813641).

## Supplementary Materials

The following supporting information can be downloaded at: https://www.mdpi.com/article/10.3390/medicina62071352/s1, Table S1: Country-specific age-standardized diabetes incidence rates across 21 MENA countries, 1990 and 2023; Table S2: Country-specific age-standardized diabetes prevalence rates across 21 MENA countries, 1990 and 2023; Table S3: Country-specific age-standardized diabetes mortality rates across 21 MENA countries, 1990 and 2023; Table S4: Country-specific age-standardized diabetes DALY rates across 21 MENA countries, 1990 and 2023; Table S5: Absolute numbers of diabetes incidence, prevalence, mortality, and DALYs across 21 MENA countries, 1990 and 2023; Table S6: Country-specific age-standardized diabetes burden (incidence, prevalence, mortality, and DALYs) across 21 MENA countries, 2023; Table S7: Comparison of pre-2019 and post-2019 average annual percent changes in diabetes burden across 21 MENA countries; Table S8: Risk-factor-attributable age-standardized diabetes DALY rates (per 100,000; 95% UI) across 21 MENA countries, 2023; Table S9: Disability composition (YLD/YLL fractions) of diabetes DALYs across 21 MENA countries, 2023; Figure S1: Male-to-female ratio of age-specific diabetes DALY rates in the MENA region, 1990 versus 2023. Figure S2: Joinpoint-fitted trends in age-standardized diabetes DALY rates for the remaining 16 MENA countries, 1990–2023. Figure S3: Country-specific average annual percent changes in age-standardized diabetes burden, MENA region, 1990–2023. Checklist S1. GATHER checklist. Checklist S2. RECORD-STROBE checklist for observational studies using routinely collected health data.

## Abbreviations

The following abbreviations are used in this manuscript:

AAPC	Average annual percent change
APC	Annual percent change
ASR	Age-standardized rate
BMI	Body-mass index
CI	Confidence interval
COVID-19	Coronavirus disease 2019
DALY	Disability-adjusted life-year
GBD	Global Burden of Disease
GCC	Gulf Cooperation Council
ICD-10	International Statistical Classification of Diseases, 10th Revision
IDF	International Diabetes Federation
IHME	Institute for Health Metrics and Evaluation
MENA	Middle East and North Africa
PAF	Population attributable fraction
PM	Particulate matter pollution
T1DM	Type 1 diabetes mellitus
TMREL	Theoretical minimum-risk exposure level
UAE	United Arab Emirates
UI	Uncertainty interval
YLD	Years lived with disability
YLL	Years of life lost

## References

[1] International Diabetes Federation (2025). IDF Diabetes Atlas.

[2] Genitsaridi I., Salpea P., Salim A., Sajjadi S.F., Tomic D., James S., Thirunavukkarasu S., Issaka A., Chen L., Basit A. (2025). 11th Edition of the IDF Diabetes Atlas: Global, Regional, and National Diabetes Prevalence Estimates for 2024 and Projections for 2050. Lancet Diabetes Endocrinol..

[3] GBD 2021 Diabetes Collaborators (2023). Global, Regional, and National Burden of Diabetes from 1990 to 2021, with Projections of Prevalence to 2050: A Systematic Analysis for the Global Burden of Disease Study 2021. Lancet.

[4] Afshin A., Sur P.J., Fay K.A., Cornaby L., Ferrara G., Salama J.S., Mullany E.C., Abate K.H., Abbafati C., Abebe Z. (2019). Health Effects of Dietary Risks in 195 Countries, 1990–2017: A Systematic Analysis for the Global Burden of Disease Study 2017. Lancet.

[5] Khalil A.B., Beshyah S.A., Abdella N., Afandi B., Al-Arouj M.M., Benbarka M.M., Al-Awadi F., Benbarka M., Ben Nakhi A., Fiad T.M. (2018). Diabesity in the Arabian Gulf: Challenges and Opportunities. Oman Med. J..

[6] NCD Risk Factor Collaboration (NCD-RisC) (2017). Worldwide Trends in Body-Mass Index, Underweight, Overweight, and Obesity from 1975 to 2016: A Pooled Analysis of 2416 Population-Based Measurement Studies in 128.9 Million Children, Adolescents, and Adults. Lancet.

[7] NCD Risk Factor Collaboration (NCD-RisC) (2024). Worldwide Trends in Underweight and Obesity from 1990 to 2022: A Pooled Analysis of 3663 Population-Representative Studies with 222 Million Children, Adolescents, and Adults. Lancet.

[8] GBD 2019 Tobacco Collaborators (2021). Spatial, Temporal, and Demographic Patterns in Prevalence of Smoking Tobacco Use and Attributable Disease Burden in 204 Countries and Territories, 1990–2019: A Systematic Analysis from the Global Burden of Disease Study 2019. Lancet.

[9] Strain T., Flaxman S., Guthold R., Semenova E., Cowan M., Riley L.M., Bull F.C., Stevens G.A., Raheem R.A., Agoudavi K. (2024). National, Regional, and Global Trends in Insufficient Physical Activity Among Adults from 2000 to 2022: A Pooled Analysis of 507 Population-Based Surveys with 5.7 Million Participants. Lancet Glob. Health.

[10] Mokdad A.H., Forouzanfar M.H., Daoud F., El Bcheraoui C., Moradi-Lakeh M., Khalil I., Afshin A., Tuffaha M., Charara R., Barber R.M. (2016). Health in Times of Uncertainty in the Eastern Mediterranean Region, 1990–2013: A Systematic Analysis for the Global Burden of Disease Study 2013. Lancet Glob. Health.

[11] Mokdad A.H., Jaber S., Aziz M.I.A., AlBuhairan F., AlGhaithi A., AlHamad N.M., Al-Hooti S.N., Al-Jasari A., AlMazroa M.A., AlQasmi A.M. (2014). The State of Health in the Arab World, 1990–2010: An Analysis of the Burden of Diseases, Injuries, and Risk Factors. Lancet.

[12] Xie J., Wang M., Long Z., Ning H., Li J., Cao Y., Liao Y., Liu G., Wang F., Pan A. (2022). Global Burden of Type 2 Diabetes in Adolescents and Young Adults, 1990–2019: Systematic Analysis of the Global Burden of Disease Study 2019. BMJ.

[13] Muller J.A., Gross R., Conzelmann C., Kruger J., Merle U., Steinhart J., Weil T., Koepke L., Bozzo C.P., Read C. (2021). SARS-CoV-2 Infects and Replicates in Cells of the Human Endocrine and Exocrine Pancreas. Nat. Metab..

[14] Stockwell S., Trott M., Tully M., Shin J., Barnett Y., Butler L., McDermott D., Schuch F., Smith L. (2021). Changes in Physical Activity and Sedentary Behaviours from Before to During the COVID-19 Pandemic Lockdown: A Systematic Review. BMJ Open Sport Exerc. Med..

[15] Kiarie H., Temmerman M., Nyamai M., Liku N., Thuo W., Oramisi V., Nyaga L., Karimi J., Wamalwa P., Gatheca G. (2022). The COVID-19 Pandemic and Disruptions to Essential Health Services in Kenya: A Retrospective Time-Series Analysis. Lancet Glob. Health.

[16] World Health Organization (2020). Pulse Survey on Continuity of Essential Health Services During the COVID-19 Pandemic: Interim Report.

[17] Kautzky-Willer A., Harreiter J., Pacini G. (2016). Sex and Gender Differences in Risk, Pathophysiology and Complications of Type 2 Diabetes Mellitus. Endocr. Rev..

[18] Stevens G.A., Alkema L., Black R.E., Boerma J.T., Collins G.S., Ezzati M., Grove J.T., Hogan D.R., Hogan M.C., Horton R. (2016). Guidelines for Accurate and Transparent Health Estimates Reporting: The GATHER Statement. Lancet.

[19] von Elm E., Altman D.G., Egger M., Pocock S.J., Gotzsche P.C., Vandenbroucke J.P. (2007). The Strengthening the Reporting of Observational Studies in Epidemiology (STROBE) Statement: Guidelines for Reporting Observational Studies. PLoS Med..

[20] World Health Organization *International Statistical Classification of Diseases and Related Health Problems, 10th Revision*, 2019 Version. https://icd.who.int/browse10/2019/en.

[21] GBD 2023 Collaborators (2025). Burden of 375 Diseases and Injuries, Risk-Attributable Burden of 88 Risk Factors, and Healthy Life Expectancy in 204 Countries and Territories, Including 660 Subnational Locations, 1990–2023: A Systematic Analysis for the Global Burden of Disease Study 2023. Lancet.

[22] GBD 2019 Diseases and Injuries Collaborators (2020). Global Burden of 369 Diseases and Injuries in 204 Countries and Territories, 1990–2019: A Systematic Analysis for the Global Burden of Disease Study 2019. Lancet.

[23] Kim H.J., Fay M.P., Feuer E.J., Midthune D.N. (2000). Permutation Tests for Joinpoint Regression with Applications to Cancer Rates. Stat. Med..

[24] Safiri S., Karamzad N., Kaufman J.S., Bell A.W., Nejadghaderi S.A., Sullman M.J.M., Moradi-Lakeh M., Collins G., Kolahi A.-A. (2022). Prevalence, Deaths and Disability-Adjusted-Life-Years (DALYs) Due to Type 2 Diabetes and Its Attributable Risk Factors in 204 Countries and Territories, 1990–2019: Results from the Global Burden of Disease Study 2019. Front. Endocrinol..

[25] Wahabi H., Esmaeil S., Zeidan R., Jamal A., Fayed A.A. (2023). Age and Gender-Specific Pattern of Cardiovascular Disease Risk Factors in Saudi Arabia: A Subgroup Analysis from the Heart Health Promotion Study. Healthcare.

[26] Bryazka D., Reitsma M.B., Abate Y.H., Abbafati C., Abbasi-Kangevari M., Abdelalim A., Abdelkader A., Abdollahi A., Abdoun M., Abdulkader R.S. (2024). Forecasting the Effects of Smoking Prevalence Scenarios on Years of Life Lost and Life Expectancy from 2022 to 2050: A Systematic Analysis for the Global Burden of Disease Study 2021. Lancet Public Health.

[27] Abdulrahim S., Jawad M. (2018). Socioeconomic Differences in Smoking in Jordan, Lebanon, Syria, and Palestine: A Cross-Sectional Analysis of National Surveys. PLoS ONE.

[28] Osabi L.A., van de Klundert J., Alhurishi S.A., Cramm J.M. (2023). A Theory-Informed Systematic Review to Understand Physical Activity Among Women in Gulf Cooperation Council Countries. BMC Public Health.

[29] Bhutta Z.A., Salam R.A., Gomber A., Lewis-Watts L., Narang T., Mbanya J.C., Alleyne G. (2021). A Century Past the Discovery of Insulin: Global Progress and Challenges for Type 1 Diabetes Among Children and Adolescents in Low-Income and Middle-Income Countries. Lancet.

[30] Xie Y., Al-Aly Z. (2022). Risks and Burdens of Incident Diabetes in Long COVID: A Cohort Study. Lancet Diabetes Endocrinol..

[31] Ssentongo P., Zhang Y., Witmer L., Chinchilli V.M., Ba D.M. (2022). Association of COVID-19 with Diabetes: A Systematic Review and Meta-Analysis. Sci. Rep..

[32] Zhou J., Wang Y., Xu J. (2024). Association of COVID-19 Infection and the Risk of New Incident Diabetes: A Systematic Review and Meta-Analysis. Front. Endocrinol..

[33] Belkhadir J. (2020). COVID-19 and Diabetes from IDF MENA Region. Diabetes Res. Clin. Pract..

[34] Hammerich A., Fouad H., Elrayah E.E., Slama S., El-Awa F., El-Berri H., Latif N.A. (2022). The Impact of the COVID-19 Pandemic on Service Delivery for Noncommunicable Diseases in the Eastern Mediterranean Region. East. Mediterr. Health J..

[35] Alkhalawi E., Vasung V., Hammerich A., Hajjar M., El-Adawy M. (2025). Maintaining Essential Non-Communicable Disease Services in the Eastern Mediterranean Region During the COVID-19 Pandemic. East. Mediterr. Health J..

[36] Alshareefy Y. (2023). The Promise of Peace Talks for Yemen’s Health-Care System. Lancet.

[37] Kehlenbrink S., Smith J., Ansbro E., Fuhr D.C., Cheung A., Ratnayake R., Boulle P., Jobanputra K., Perel P., Roberts B. (2019). The Burden of Diabetes and Use of Diabetes Care in Humanitarian Crises in Low-Income and Middle-Income Countries. Lancet Diabetes Endocrinol..

[38] Devi S. (2021). Yemen’s Health System Has “Collapsed”, Warns UN. Lancet.

[39] World Bank Urban Population (% of Total Population). https://data.worldbank.org/indicator/SP.URB.TOTL.IN.ZS.

[40] Alawainati M., AlGhareeb N., Najem W., Albulushi B., Alshaikh A., Al-Tajer D., Buhejji M., Almulla A., Obaid S., AlRabiah J. (2025). Epidemiology of Macrovascular and Microvascular Complications Among Patients with Diabetes Mellitus in Primary Care in Bahrain. Cureus.

[41] Yang B.Y., Fan S., Thiering E., Seissler J., Nowak D., Dong G.H., Heinrich J. (2020). Ambient Air Pollution and Diabetes: A Systematic Review and Meta-Analysis. Environ. Res..

[42] Colchero M.A., Popkin B.M., Rivera J.A., Ng S.W. (2016). Beverage Purchases from Stores in Mexico Under the Excise Tax on Sugar Sweetened Beverages: Observational Study. BMJ.

[43] Teng A.M., Jones A.C., Mizdrak A., Signal L., Genc M., Wilson N. (2019). Impact of Sugar-Sweetened Beverage Taxes on Purchases and Dietary Intake: Systematic Review and Meta-Analysis. Obes. Rev..

[44] Al-Jawaldeh A., Perucic A.M., Hammerich A., Abaal D.I., Alhajjeh A.S., Abed Y., Ibrahim E.T., AlMatrooshi F.E., Alkhalaf M.M., Letaief M. (2024). A Review of Sugar-Sweetened Beverages Taxation in Saudi Arabia and United Arab Emirates. East. Mediterr. Health J..

