# Supplementary material for: Diabetes Burden in the Middle East and North Africa Region, 1990–2023: An Ecological Time-Trend Analysis of GBD Estimates
Source: Medicina (Kaunas). 2026 Jul 13;62(7):1352. doi: 10.3390/medicina62071352 (PMC13414397; doi:10.3390/medicina62071352)
Supplement: Supplementary file 1 [file medicina-62-01352-s001.zip › medicina-4383699-supplementary.pdf]

SUPPLEMENTARY MATERIALS

Table S1. Country-specific age-standardized diabetes incidence rates across 21 MENA countries, 1990 and 2023.
Table S2. Country-specific age-standardized diabetes prevalence rates across 21 MENA countries, 1990 and 2023.
Table S3. Country-specific age-standardized diabetes mortality rates across 21 MENA countries, 1990 and 2023.
Table S4. Country-specific age-standardized diabetes DALY rates across 21 MENA countries, 1990 and 2023.
Table S5. Absolute numbers of diabetes incidence, prevalence, mortality, and DALYs across 21 MENA countries, 1990 and 2023.
Table S6. Country-specific age-standardized diabetes burden (incidence, prevalence, mortality, and DALYs) across 21 MENA countries, 2023.
Table S7. Comparison of pre-2019 and post-2019 average annual percent changes in diabetes burden across 21 MENA countries.
Table S8. Risk-factor-attributable age-standardized diabetes DALY rates (per 100,000; 95% UI) across 21 MENA countries, 2023.
Table S9. Disability composition (YLD/YLL fractions) of diabetes DALYs across 21 MENA countries, 2023.

Figure S1. Male-to-female ratio of age-specific diabetes DALY rates in the MENA region, 1990 versus 2023.
Figure S2. Joinpoint-fitted trends in age-standardized diabetes DALY rates for the remaining 16 MENA countries, 1990–2023.
Figure S3. Country-specific average annual percent changes in age-standardized diabetes burden, MENA region, 1990–2023.

Checklist S1. GATHER checklist.
Checklist S2. RECORD-STROBE checklist for observational studies using routinely collected health data.

Table S1. Age-standardized incidence rates (per 100,000 population) and Average Annual Percent Change (AAPC) for diabetes mellitus across 21 Middle East and North Africa countries, 1990 and 2023.

Country	1990 Rate (95% UI) (per 100k)	2023 Rate (95% UI) (per 100k)	% Change	AAPC 1990-2023 (%)	P-value AAPC
**Middle East and North Africa**	251.7 (231.5–272.4)	482.5 (451.5–516.4)	91.70%	1.99 (1.97–2.02)	<0.001
**Afghanistan**	267.1 (240.3–290.9)	420.4 (385.2–454.4)	57.40%	1.39 (1.38–1.41)	<0.001
**Algeria**	263.5 (239.2–287.8)	480.6 (443.2–516.0)	82.40%	1.84 (1.80–1.88)	<0.001
**Bahrain**	462.3 (430.7–493.1)	770.2 (710.1–823.4)	66.60%	1.54 (1.51–1.57)	<0.001
**Egypt**	252.1 (230.6–273.6)	590.8 (551.1–633.8)	134.40%	2.61 (2.60–2.63)	<0.001
**Iran**	192.8 (172.8–212.4)	355.4 (320.9–391.4)	84.30%	1.87 (1.81–1.91)	<0.001
**Iraq**	304.1 (277.3–327.7)	550.4 (507.2–593.6)	81.00%	1.81 (1.79–1.83)	<0.001
**Jordan**	364.0 (340.7–387.4)	535.4 (499.6–568.0)	47.10%	1.17 (1.15–1.19)	<0.001
**Kuwait**	453.7 (424.1–491.7)	757.0 (702.9–809.1)	66.90%	1.56 (1.55–1.58)	<0.001
**Lebanon**	292.1 (265.1–321.8)	499.0 (460.3–538.2)	70.80%	1.64 (1.63–1.65)	<0.001
**Libya**	267.1 (240.6–291.2)	553.8 (509.2–597.9)	107.30%	2.25 (2.21–2.28)	<0.001
**Morocco**	236.3 (212.9–257.7)	411.4 (373.7–443.5)	74.10%	1.70 (1.68–1.71)	<0.001
**Oman**	268.8 (245.3–288.6)	471.5 (436.2–511.1)	75.40%	1.71 (1.70–1.72)	<0.001
**Palestine**	316.3 (294.4–342.6)	547.6 (506.9–594.8)	73.10%	1.67 (1.64–1.70)	<0.001
**Qatar**	400.4 (368.5–436.3)	632.4 (586.0–689.9)	57.90%	1.40 (1.37–1.42)	<0.001
**Saudi Arabia**	456.1 (417.5–493.2)	899.4 (839.2–963.5)	97.20%	2.08 (2.06–2.11)	<0.001
**Sudan**	204.2 (183.1–221.3)	366.9 (336.2–397.1)	79.70%	1.78 (1.76–1.80)	<0.001
**Syria**	267.8 (244.4–294.0)	520.0 (477.9–560.7)	94.20%	2.04 (2.03–2.05)	<0.001
**Türkiye**	234.3 (216.4–253.7)	374.9 (344.5–409.4)	60.00%	1.32 (1.21–1.44)	<0.001
**Tunisia**	294.8 (270.7–319.9)	449.4 (416.3–486.0)	52.40%	1.28 (1.27–1.30)	<0.001
**United Arab Emirates**	386.4 (350.6–420.6)	601.9 (556.7–646.1)	55.80%	1.35 (1.33–1.36)	<0.001
**Yemen**	128.5 (115.4–140.0)	307.5 (277.5–338.1)	139.30%	2.69 (2.67–2.72)	<0.001

Table S2. Age-standardized prevalence rates (per 100,000 population) and Average Annual Percent Change (AAPC) for diabetes mellitus across 21 Middle East and North Africa countries, 1990 and 2023.

Country	Age-standardized Prev Rate 1990 (per 100k) (95% UI)	Age-standardized Prev Rate 2023 (per 100k) (95% UI)	% Change in Rate	AAPC 1990-2023 (%)	P-value
**MENA**	5564.13 (5088.28, 6023.84)	11247.15 (10381.47, 12131.77)	102.10%	2.15 (2.13-2.16)	< 0.001
**Afghanistan**	6153.28 (5527.71, 6755.34)	10383.21 (9478.59, 11275.07)	68.70%	1.61 (1.60-1.62)	< 0.001
**Algeria**	5935.58 (5333.62, 6501.07)	11163.01 (10304.01, 12073.55)	88.10%	1.93 (1.91-1.95)	< 0.001
**Bahrain**	9952.39 (9107.94, 10793.07)	18377.71 (16881.72, 19690.80)	84.70%	1.86 (1.84-1.89)	< 0.001
**Egypt**	5531.56 (5021.08, 6015.99)	13952.79 (12943.78, 15091.37)	152.20%	2.84 (2.84-2.85)	< 0.001
**Iran**	4432.82 (3979.85, 4892.09)	8241.80 (7382.02, 9142.33)	85.90%	1.89 (1.84-1.94)	< 0.001
**Iraq**	6840.11 (6207.14, 7439.93)	13229.22 (12066.07, 14381.47)	93.40%	2.01 (1.99-2.03)	< 0.001
**Jordan**	7959.95 (7370.43, 8464.56)	12579.46 (11726.40, 13397.34)	58.00%	1.39 (1.36-1.42)	< 0.001
**Kuwait**	10241.27 (9471.99, 11123.85)	18530.72 (17124.38, 20059.78)	80.90%	1.81 (1.79-1.83)	< 0.001
**Lebanon**	6736.43 (6161.17, 7416.13)	12323.33 (11305.64, 13324.77)	82.90%	1.85 (1.83-1.88)	< 0.001
**Libya**	6226.36 (5563.99, 6846.52)	13781.17 (12589.95, 15156.28)	121.30%	2.45 (2.41-2.48)	< 0.001
**Morocco**	5414.76 (4874.74, 5941.35)	9860.00 (9074.27, 10628.61)	82.10%	1.83 (1.82-1.84)	< 0.001
**Oman**	5786.01 (5279.03, 6242.01)	11070.36 (10182.41, 12040.29)	91.30%	1.98 (1.97-2.00)	< 0.001
**Palestine**	6709.27 (6134.40, 7294.48)	12703.89 (11642.73, 13798.18)	89.30%	1.95 (1.92-1.97)	< 0.001
**Qatar**	9198.47 (8339.02, 9997.47)	15297.87 (14107.76, 16800.91)	66.30%	1.56 (1.53-1.58)	< 0.001
**Saudi Arabia**	10648.03 (9629.05, 11634.72)	23138.32 (21309.04, 25001.54)	117.30%	2.37 (2.33-2.40)	< 0.001
**Sudan**	4617.91 (4168.43, 5045.47)	8722.60 (8022.63, 9549.17)	88.90%	1.94 (1.91-1.98)	< 0.001
**Syria**	6058.95 (5519.34, 6676.22)	12676.27 (11539.37, 13840.93)	109.20%	2.27 (2.26-2.29)	< 0.001
**Tunisia**	6443.21 (5881.65, 7001.26)	10362.43 (9520.43, 11264.88)	60.80%	1.45 (1.44-1.46)	< 0.001
**Türkiye**	4989.47 (4527.84, 5432.60)	8599.46 (7815.93, 9433.65)	72.40%	1.61 (1.43-1.79)	< 0.001
**United Arab Emirates**	9010.67 (8192.14, 9764.63)	15752.52 (14421.42, 17114.01)	74.80%	1.70 (1.68-1.72)	< 0.001
**Yemen**	2733.77 (2463.63, 2991.92)	7298.84 (6509.56, 7995.36)	167.00%	3.03 (3.01-3.06)	< 0.001

Table S3. Country-specific age-standardized mortality rates for diabetes mellitus, 1990 and 2023.

Country	Age-standardized Rate 1990 (per 100k)	Age-standardized Rate 2023 (per 100k)	% Change in Rates	AAPC 1990-2023 (%)	P-value
**MENA**	26.90 (17.48, 37.95)	30.58 (24.75, 36.83)	13.70%	0.37 (0.30-0.47)	< 0.001
**Afghanistan**	21.89 (11.14, 37.38)	26.99 (15.32, 41.29)	23.30%	0.64 (0.58-0.71)	< 0.001
**Algeria**	18.46 (9.71, 33.32)	23.95 (13.91, 36.61)	29.70%	0.89 (0.73-1.08)	< 0.001
**Bahrain**	101.70 (74.42, 131.88)	116.98 (88.40, 146.89)	15.00%	0.52 (0.26-0.79)	< 0.001
**Egypt**	31.41 (20.35, 49.51)	48.40 (36.35, 63.26)	54.10%	1.29 (0.98-1.61)	< 0.001
**Iran**	14.01 (9.34, 19.24)	20.38 (14.79, 26.39)	45.50%	1.16 (0.74-1.50)	< 0.001
**Iraq**	45.67 (27.79, 68.64)	59.75 (41.05, 79.55)	30.80%	0.85 (0.64-1.03)	< 0.001
**Jordan**	55.02 (38.48, 71.61)	34.25 (27.55, 42.35)	-37.70%	-1.42 (-1.60--1.24)	< 0.001
**Kuwait**	37.08 (33.07, 41.07)	37.54 (32.51, 42.79)	1.20%	0.10 (-0.71-1.07)	0.653
**Lebanon**	13.94 (8.68, 20.44)	10.90 (8.43, 14.12)	-21.80%	-0.76 (-1.22--0.30)	< 0.001
**Libya**	18.84 (9.91, 34.21)	20.32 (11.84, 33.01)	7.90%	0.18 (0.02-0.38)	< 0.001
**Morocco**	17.41 (8.96, 30.23)	20.97 (12.12, 32.97)	20.40%	0.56 (0.48-0.64)	< 0.001
**Oman**	46.19 (31.02, 61.57)	56.47 (40.21, 72.77)	22.30%	0.59 (0.35-0.76)	< 0.001
**Palestine**	47.03 (34.30, 62.80)	58.57 (43.65, 70.25)	24.50%	0.63 (0.39-0.90)	< 0.001
**Qatar**	83.07 (60.45, 108.63)	49.72 (37.97, 64.08)	-40.10%	-1.57 (-2.04--1.15)	< 0.001
**Saudi Arabia**	25.26 (16.91, 35.27)	58.74 (43.50, 74.29)	132.50%	2.57 (2.36-2.77)	< 0.001
**Sudan**	16.99 (8.63, 29.70)	21.30 (12.66, 33.77)	25.40%	0.67 (0.60-0.76)	< 0.001
**Syria**	22.95 (15.91, 30.79)	22.58 (16.00, 31.56)	-1.60%	-0.10 (-0.47-0.12)	0.104
**Tunisia**	68.83 (45.24, 96.82)	52.06 (41.61, 65.92)	-24.40%	-0.79 (-1.10--0.40)	< 0.001
**Türkiye**	32.82 (23.01, 44.30)	27.85 (21.12, 34.87)	-15.20%	-0.50 (-0.69--0.28)	< 0.001
**United Arab Emirates**	65.07 (41.31, 96.25)	30.76 (22.07, 41.43)	-52.70%	-2.18 (-2.42--1.89)	< 0.001
**Yemen**	17.87 (8.82, 31.13)	19.29 (11.92, 29.45)	7.90%	0.22 (0.11-0.35)	< 0.001

Table S4. Age-standardized DALY rates (per 100,000 population) and Average Annual Percent Change (AAPC) for diabetes mellitus across 21 Middle East and North Africa countries, 1990 and 2023.

Country	1990 Rate (95% UI) (per 100k)	2023 Rate (95% UI) (per 100k)	% Change	AAPC 1990-2023 (%)	P-value
**MENA**	1007.8 (759.5–1267.7)	1491.8 (1180.9–1807.0)	48.00%	1.18 (1.13–1.21)	<0.001
**Afghanistan**	953.7 (666.9–1325.1)	1373.1 (1014.5–1727.5)	44.00%	1.11 (1.03–1.19)	<0.001
**Algeria**	844.2 (578.5–1163.4)	1345.2 (1014.7–1715.9)	59.40%	1.46 (1.38–1.54)	<0.001
**Bahrain**	2762.3 (2213.3–3322.4)	3460.6 (2807.8–4214.6)	25.30%	0.66 (0.58–0.75)	<0.001
**Egypt**	1155.4 (863.7–1526.7)	2082.4 (1660.0–2505.5)	80.20%	1.88 (1.68–2.03)	<0.001
**Iran**	669.1 (514.8–826.1)	1081.1 (845.3–1365.7)	61.60%	1.48 (1.37–1.60)	<0.001
**Iraq**	1554.1 (1141.2–1997.5)	2261.3 (1851.2–2705.3)	45.50%	1.18 (1.10–1.34)	<0.001
**Jordan**	1746.9 (1390.2–2165.4)	1557.4 (1202.9–1931.3)	-10.80%	-0.35 (-0.42–-0.29)	<0.001
**Kuwait**	1508.1 (1239.6–1805.4)	2053.1 (1620.7–2630.4)	36.10%	0.96 (0.62–1.24)	<0.001
**Lebanon**	877.0 (661.7–1107.7)	1277.5 (949.3–1653.5)	45.70%	1.17 (1.09–1.26)	<0.001
**Libya**	904.8 (616.1–1262.7)	1493.3 (1115.2–1921.0)	65.00%	1.52 (1.44–1.62)	<0.001
**Morocco**	802.3 (555.9–1089.2)	1181.4 (869.4–1491.6)	47.30%	1.17 (1.12–1.22)	<0.001
**Oman**	1451.9 (1108.9–1805.1)	1911.7 (1486.5–2383.5)	31.70%	0.81 (0.67–0.92)	<0.001
**Palestine**	1485.7 (1182.2–1862.0)	2104.6 (1711.8–2529.9)	41.70%	1.05 (0.87–1.30)	<0.001
**Qatar**	2191.1 (1733.7–2653.2)	2002.4 (1574.7–2450.0)	-8.60%	-0.24 (-0.60–0.03)	> 0.05
**Saudi Arabia**	1243.9 (975.4–1544.5)	2648.6 (2112.6–3280.2)	112.90%	2.31 (2.24–2.39)	<0.001
**Sudan**	699.5 (480.9–985.1)	1068.0 (771.0–1407.5)	52.70%	1.29 (1.23–1.36)	<0.001
**Syria**	939.1 (721.0–1159.4)	1384.0 (1033.9–1765.6)	47.40%	1.17 (1.03–1.31)	<0.001
**Türkiye**	1093.7 (852.8–1333.7)	1262.2 (968.7–1551.2)	15.40%	0.41 (0.36–0.46)	<0.001
**Tunisia**	1791.6 (1331.3–2299.0)	1803.5 (1448.6–2179.3)	0.70%	0.07 (-0.13–0.32)	0.102
**United Arab Emirates**	2013.7 (1493.3–2668.2)	1581.9 (1200.3–2000.4)	-21.40%	-0.71 (-0.83–-0.58)	<0.001
**Yemen**	603.7 (396.3–863.2)	973.7 (727.5–1255.4)	61.30%	1.45 (1.41–1.51)	<0.001

Table S5. Absolute numbers (in thousands; 95% UI) and percentage change for diabetes incidence, prevalence, mortality, and DALYs in 21 Middle East and North Africa countries and the region overall, 1990 and 2023.

Location	Measure	Number in 1990, in thousands (95% UI)	Number in 2023, in thousands (95% UI)	Percentage Change, % (95% UI)
**Middle East and North Africa**	Incidence	644.2 (592.6-693.1)	3,188.3 (2962.6-3408.0)	394.9 (327.5-475.1)
	Prevalence	11,720.5 (10689.7-12800.5)	66,580.4 (61322.9-72110.3)	468.1 (379.1-574.6)
	Mortality	38.1 (25.4-53.4)	127.2 (104.5-152.4)	234.0 (95.7-501.0)
	DALYs	1,898.7 (1452.7-2356.8)	8,024.0 (6278.1-9804.3)	322.6 (166.4-574.9)
**Afghanistan**	Incidence	21.4 (19.1-23.3)	118.2 (107.7-129.6)	453.1 (361.4-578.2)
	Prevalence	455.9 (408.0-500.7)	2,034.3 (1856.4-2231.4)	346.2 (270.7-446.9)
	Mortality	1.3 (0.7-2.3)	2.8 (1.7-4.1)	107.6 (-28.4-495.9)
	DALYs	71.4 (50.0-99.5)	229.1 (172.4-286.7)	220.9 (73.4-473.7)
**Algeria**	Incidence	48.5 (43.9-53.0)	224.2 (204.7-241.6)	362.4 (286.1-450.3)
	Prevalence	894.1 (805.3-978.6)	4,894.8 (4517.9-5314.8)	447.4 (361.6-560.0)
	Mortality	1.9 (1.0-3.4)	8.2 (4.8-12.5)	326.8 (40.9-1112.7)
	DALYs	118.4 (82.1-161.4)	557.8 (420.6-711.1)	371.0 (160.5-766.6)
**Bahrain**	Incidence	1.9 (1.8-2.2)	16.0 (14.5-17.5)	724.7 (571.6-888.0)
	Prevalence	30.7 (28.0-33.4)	306.9 (281.1-333.5)	898.7 (740.6-1091.4)
	Mortality	0.1 (0.1-0.2)	0.7 (0.6-0.9)	456.5 (235.7-852.7)
	DALYs	5.6 (4.5-6.7)	40.7 (31.9-50.0)	623.4 (373.6-1001.5)
**Egypt**	Incidence	109.9 (99.9-118.5)	609.8 (569.7-655.6)	454.7 (380.7-556.5)
	Prevalence	1,965.3 (1768.5-2141.6)	12,216.9 (11265.0-13242.4)	521.6 (426.0-648.8)
	Mortality	7.3 (5.1-10.9)	26.0 (20.0-34.0)	254.1 (82.9-564.6)
	DALYs	362.0 (275.6-463.6)	1,590.3 (1235.5-1967.6)	339.4 (166.5-614.1)
**Iran**	Incidence	78.8 (70.6-86.5)	363.2 (323.9-403.2)	361.1 (274.6-470.9)
	Prevalence	1,475.8 (1324.9-1635.5)	7,893.6 (7064.6-8750.2)	434.9 (332.0-560.4)
	Mortality	3.2 (2.2-4.3)	14.2 (10.4-18.4)	346.7 (139.5-745.5)
	DALYs	205.9 (160.0-255.4)	959.9 (738.5-1225.8)	366.1 (189.2-666.2)
**Iraq**	Incidence	38.9 (35.4-42.0)	231.3 (212.8-250.1)	493.7 (406.4-606.7)
	Prevalence	686.8 (621.4-749.8)	4,496.4 (4081.9-4880.8)	554.7 (444.4-685.5)
	Mortality	3.3 (2.1-4.9)	12.6 (9.1-16.2)	276.5 (85.5-664.5)
	DALYs	140.3 (106.5-175.9)	655.3 (523.5-784.4)	367.1 (197.6-636.2)
**Jordan**	Incidence	9.0 (8.4-9.5)	73.1 (68.2-77.8)	714.7 (615.9-826.8)
	Prevalence	147.5 (136.1-157.4)	1,409.2 (1305.5-1515.4)	855.5 (729.3-1013.7)
	Mortality	0.6 (0.5-0.8)	2.3 (1.9-2.8)	258.2 (127.4-509.3)
	DALYs	27.1 (21.5-32.9)	151.1 (114.2-190.0)	458.0 (246.8-783.5)
**Kuwait**	Incidence	6.8 (6.2-7.5)	45.9 (42.3-49.6)	577.2 (467.8-701.6)
	Prevalence	103.6 (93.2-114.7)	929.4 (853.1-1017.8)	797.2 (643.6-991.6)
	Mortality	0.1 (0.1-0.1)	0.6 (0.6-0.7)	439.3 (338.1-576.0)
	DALYs	10.4 (8.1-12.9)	83.1 (60.8-109.6)	701.3 (370.7-1257.1)
**Lebanon**	Incidence	8.2 (7.5-9.0)	29.9 (27.4-32.2)	264.4 (204.5-331.1)
	Prevalence	169.4 (154.2-186.1)	747.8 (686.4-808.7)	341.6 (268.8-424.4)
	Mortality	0.3 (0.2-0.4)	0.7 (0.5-0.9)	131.2 (22.3-380.5)
	DALYs	20.9 (15.8-26.6)	76.8 (56.9-99.3)	266.7 (114.2-527.5)
**Libya**	Incidence	8.3 (7.5-9.1)	47.3 (42.9-51.7)	467.3 (370.5-589.4)
	Prevalence	150.4 (135.3-165.1)	1,028.5 (934.7-1134.8)	583.8 (466.3-738.8)
	Mortality	0.3 (0.2-0.6)	1.0 (0.6-1.7)	210.8 (1.3-826.8)
	DALYs	19.9 (13.8-27.4)	102.7 (76.6-132.8)	417.3 (179.5-863.4)
**Morocco**	Incidence	47.7 (43.1-52.0)	167.4 (151.7-180.8)	251.1 (191.6-320.0)
	Prevalence	919.4 (829.2-1011.6)	3,925.5 (3613.0-4230.5)	327.0 (257.2-410.2)
	Mortality	2.3 (1.2-3.8)	6.7 (3.9-10.5)	199.1 (1.6-805.8)
	DALYs	128.0 (88.7-172.1)	453.6 (335.9-571.9)	254.5 (95.1-544.5)
**Oman**	Incidence	3.9 (3.5-4.2)	26.9 (24.4-29.8)	592.7 (479.1-746.7)
	Prevalence	62.5 (56.8-68.4)	479.6 (432.1-524.5)	668.0 (532.0-823.8)
	Mortality	0.3 (0.2-0.4)	0.9 (0.7-1.2)	243.6 (84.5-542.9)
	DALYs	12.1 (9.4-14.9)	57.6 (44.8-71.6)	375.9 (200.4-660.8)
**Palestine**	Incidence	4.0 (3.7-4.3)	25.5 (23.4-27.5)	539.0 (443.0-644.2)
	Prevalence	69.9 (64.2-76.4)	472.6 (429.2-519.9)	576.1 (462.0-709.3)
	Mortality	0.4 (0.3-0.5)	1.3 (1.0-1.6)	253.1 (104.5-468.2)
	DALYs	14.1 (11.3-17.4)	65.0 (52.6-79.8)	360.6 (201.5-607.0)
**Qatar**	Incidence	1.7 (1.5-1.9)	25.3 (22.6-28.8)	1370.2 (1068.0-1821.8)
	Prevalence	27.1 (24.1-30.4)	452.2 (406.0-499.8)	1566.5 (1235.6-1975.7)
	Mortality	0.1 (0.0-0.1)	0.3 (0.2-0.3)	411.1 (206.0-773.0)
	DALYs	3.2 (2.5-4.0)	38.7 (28.1-49.5)	1102.7 (595.2-1894.1)
**Saudi Arabia**	Incidence	55.2 (49.6-60.1)	341.7 (309.1-382.7)	519.6 (414.3-671.7)
	Prevalence	927.3 (837.8-1010.1)	6,646.8 (6072.1-7268.7)	616.8 (501.1-767.6)
	Mortality	1.2 (0.8-1.6)	6.0 (4.6-7.5)	415.6 (184.5-830.7)
	DALYs	92.3 (71.6-115.6)	584.5 (448.8-742.7)	533.0 (288.3-936.7)
**Sudan**	Incidence	28.7 (26.1-31.2)	124.4 (113.9-134.3)	333.0 (265.1-414.8)

	Prevalence	527.4 (479.3-578.0)	2,487.3 (2289.2-2733.0)	371.7 (296.1-470.2)
	Mortality	1.4 (0.7-2.4)	4.4 (2.7-6.9)	213.8 (9.8-833.5)
	DALYs	77.2 (53.6-110.1)	279.9 (205.2-364.4)	262.4 (86.5-580.0)
**Syria**	Incidence	22.7 (20.6-24.8)	100.2 (91.2-109.5)	341.9 (268.3-431.7)
	Prevalence	401.6 (364.4-444.7)	2,196.9 (1999.3-2408.2)	447.1 (349.6-560.8)
	Mortality	1.0 (0.7-1.3)	2.6 (1.9-3.6)	167.9 (44.4-436.2)
	DALYs	55.0 (42.9-67.0)	226.0 (167.9-288.7)	310.8 (150.4-572.3)
**Tunisia**	Incidence	19.8 (18.2-21.2)	62.4 (57.8-67.6)	215.8 (172.3-271.8)
	Prevalence	373.9 (339.5-408.5)	1,480.6 (1360.8-1607.3)	296.0 (233.1-373.5)
	Mortality	2.6 (1.7-3.5)	6.4 (5.1-8.1)	149.4 (46.0-385.4)
	DALYs	89.0 (66.9-112.7)	249.9 (200.4-301.9)	180.7 (77.9-351.3)
**Türkiye**	Incidence	111.3 (102.7-119.7)	370.4 (339.7-406.8)	232.8 (183.8-296.1)
	Prevalence	2,046.7 (1858.2-2231.5)	8,754.1 (7958.0-9597.8)	327.7 (256.6-416.5)
	Mortality	9.4 (6.6-12.5)	25.9 (19.7-32.7)	176.8 (58.4-391.7)
	DALYs	394.6 (307.4-479.8)	1,272.9 (972.9-1564.6)	222.6 (102.8-409.0)
**United Arab Emirates**	Incidence	6.9 (6.1-7.7)	94.1 (82.9-106.7)	1264.6 (978.1-1660.8)
	Prevalence	102.1 (91.6-111.7)	2,083.3 (1877.2-2283.1)	1939.6 (1580.6-2392.4)
	Mortality	0.2 (0.1-0.3)	0.7 (0.5-0.9)	240.3 (71.0-578.3)
	DALYs	12.2 (9.3-15.5)	158.3 (111.9-207.6)	1194.6 (619.9-2121.7)
**Yemen**	Incidence	10.4 (9.4-11.4)	88.5 (79.2-96.9)	748.9 (596.3-930.1)
	Prevalence	176.8 (159.5-194.8)	1,582.9 (1413.3-1735.3)	795.2 (625.6-988.2)
	Mortality	0.8 (0.4-1.3)	2.5 (1.6-3.8)	235.5 (23.4-871.9)
	DALYs	37.9 (25.5-57.3)	183.4 (138.0-233.0)	383.3 (140.8-812.3)

Table S6. Country-specific age-standardized diabetes burden across 21 Middle East and North Africa countries, 2023.

Country	Incidence Rate * [95% UI]	Prevalence Rate * [95% UI]	Mortality Rate * [95% UI]	DALY Rate * [95% UI]
**Middle East and North Africa**	482.5 [451.5–516.4]	11247.1 [10381.5–12131.8]	30.6 [24.7–36.8]	1491.8 [1180.9–1807.0]
**Afghanistan**	420.4 [385.2–454.4]	10383.2 [9478.6–11275.1]	27.0 [15.3–41.3]	1373.1 [1014.5–1727.5]
**Algeria**	480.6 [443.2–516.0]	11163.0 [10304.0–12073.5]	24.0 [13.9–36.6]	1345.2 [1014.7–1715.9]
**Bahrain**	770.2 [710.1–823.4]	18377.7 [16881.7–19690.8]	117.0 [88.4–146.9]	3460.6 [2807.8–4214.6]
**Egypt**	590.8 [551.1–633.8]	13952.8 [12943.8–15091.4]	48.4 [36.3–63.3]	2082.4 [1660.0–2505.5]
**Iran**	355.4 [320.9–391.4]	8241.8 [7382.0–9142.3]	20.4 [14.8–26.4]	1081.1 [845.3–1365.7]
**Iraq**	550.4 [507.2–593.6]	13229.2 [12066.1–14381.5]	59.7 [41.1–79.5]	2261.3 [1851.2–2705.3]
**Jordan**	535.4 [499.6–568.0]	12579.5 [11726.4–13397.3]	34.3 [27.5–42.4]	1557.4 [1202.9–1931.3]
**Kuwait**	757.0 [702.9–809.1]	18530.7 [17124.4–20059.8]	37.5 [32.5–42.8]	2053.1 [1620.7–2630.4]
**Lebanon**	499.0 [460.3–538.2]	12323.3 [11305.6–13324.8]	10.9 [8.4–14.1]	1277.5 [949.3–1653.5]
**Libya**	553.8 [509.2–597.9]	13781.2 [12589.9–15156.3]	20.3 [11.8–33.0]	1493.3 [1115.2–1921.0]
**Morocco**	411.4 [373.7–443.5]	9860.0 [9074.3–10628.6]	21.0 [12.1–33.0]	1181.4 [869.4–1491.6]
**Oman**	471.5 [436.2–511.1]	11070.4 [10182.4–12040.3]	56.5 [40.2–72.8]	1911.7 [1486.5–2383.5]
**Palestine**	547.6 [506.9–594.8]	12703.9 [11642.7–13798.2]	58.6 [43.7–70.2]	2104.6 [1711.8–2529.9]
**Qatar**	632.4 [586.0–689.9]	15297.9 [14107.8–16800.9]	49.7 [38.0–64.1]	2002.4 [1574.7–2450.0]
**Saudi Arabia**	899.4 [839.2–963.5]	23138.3 [21309.0–25001.5]	58.7 [43.5–74.3]	2648.6 [2112.6–3280.2]
**Sudan**	366.9 [336.2–397.1]	8722.6 [8022.6–9549.2]	21.3 [12.7–33.8]	1068.0 [771.0–1407.5]
**Syria**	520.0 [477.9–560.7]	12676.3 [11539.4–13840.9]	22.6 [16.0–31.6]	1384.0 [1033.9–1765.6]
**Tunisia**	449.4 [416.3–486.0]	10362.4 [9520.4–11264.9]	52.1 [41.6–65.9]	1803.5 [1448.6–2179.3]
**Türkiye**	374.9 [344.5–409.4]	8599.5 [7815.9–9433.6]	27.8 [21.1–34.9]	1262.2 [968.7–1551.2]
**United Arab Emirates**	601.9 [556.7–646.1]	15752.5 [14421.4–17114.0]	30.8 [22.1–41.4]	1581.9 [1200.3–2000.4]
**Yemen**	307.5 [277.5–338.1]	7298.8 [6509.6–7995.4]	19.3 [11.9–29.4]	973.7 [727.5–1255.4]

Table S7. Country-specific AAPCs for all diabetes burden metrics by period: pre-2019 (1990–2019) and post-2019 (2019–2023).

Country	Measure	Period	AAPC (% per year, 95% CI)	P-value
**Middle East and North Africa**	DALYs	1990-2019	1.17(1.12-1.23)	< 0.001
**Middle East and North Africa**	DALYs	2019-2023	1.21(0.77-1.53)	< 0.001
**Afghanistan**	DALYs	1990-2019	1.27(1.20-1.33)	< 0.001
**Afghanistan**	DALYs	2019-2023	-0.07(-0.63-0.45)	0.595
**Algeria**	DALYs	1990-2019	1.26(1.20-1.37)	< 0.001
**Algeria**	DALYs	2019-2023	2.88(1.98-3.44)	< 0.001
**Bahrain**	DALYs	1990-2019	0.75(0.66-0.84)	< 0.001
**Bahrain**	DALYs	2019-2023	0.04(-0.07-0.13)	0.196
**Egypt**	DALYs	1990-2019	2.17(1.93-2.35)	< 0.001
**Egypt**	DALYs	2019-2023	-0.21(-1.91-1.35)	0.203
**Iran**	DALYs	1990-2019	1.43(1.33-1.55)	< 0.001
**Iran**	DALYs	2019-2023	1.87(0.87-2.90)	< 0.001
**Iraq**	DALYs	1990-2019	1.17(1.09-1.33)	< 0.001
**Iraq**	DALYs	2019-2023	1.24(0.56-1.60)	< 0.001
**Jordan**	DALYs	1990-2019	-0.30(-0.36--0.25)	< 0.001
**Jordan**	DALYs	2019-2023	-0.67(-1.26--0.23)	< 0.001
**Kuwait**	DALYs	1990-2019	1.04(0.77-1.39)	< 0.001
**Kuwait**	DALYs	2019-2023	0.39(-2.34-2.68)	0.665
**Lebanon**	DALYs	1990-2019	1.00(0.92-1.08)	< 0.001
**Lebanon**	DALYs	2019-2023	2.39(1.84-3.19)	< 0.001
**Libya**	DALYs	1990-2019	1.63(1.56-1.72)	< 0.001
**Libya**	DALYs	2019-2023	0.75(0.17-1.27)	< 0.001
**Morocco**	DALYs	1990-2019	1.34(1.29-1.39)	< 0.001
**Morocco**	DALYs	2019-2023	0.00(-0.41-0.37)	0.947
**Oman**	DALYs	1990-2019	0.79(0.69-0.89)	< 0.001
**Oman**	DALYs	2019-2023	0.95(0.33-1.74)	< 0.001
**Palestine**	DALYs	1990-2019	1.04(0.95-1.28)	< 0.001
**Palestine**	DALYs	2019-2023	1.17(-0.42-1.94)	> 0.05
**Qatar**	DALYs	1990-2019	-0.33(-0.64--0.01)	< 0.001
**Qatar**	DALYs	2019-2023	0.45(-1.75-2.83)	0.328
**Saudi Arabia**	DALYs	1990-2019	2.24(2.19-2.32)	< 0.001
**Saudi Arabia**	DALYs	2019-2023	2.84(2.22-3.30)	< 0.001
**Sudan**	DALYs	1990-2019	1.32(1.27-1.39)	< 0.001
**Sudan**	DALYs	2019-2023	1.07(0.59-1.48)	< 0.001
**Syria**	DALYs	1990-2019	1.16(1.06-1.27)	< 0.001
**Syria**	DALYs	2019-2023	1.26(0.37-2.08)	< 0.001
**Tunisia**	DALYs	1990-2019	-0.16(-0.32-0.09)	> 0.05
**Tunisia**	DALYs	2019-2023	1.74(0.24-3.13)	< 0.001
**Türkiye**	DALYs	1990-2019	0.10(0.05-0.15)	< 0.001
**Türkiye**	DALYs	2019-2023	2.71(2.29-3.08)	< 0.001
**United Arab Emirates**	DALYs	1990-2019	-1.14(-1.28--0.97)	< 0.001
**United Arab Emirates**	DALYs	2019-2023	2.47(1.27-3.51)	< 0.001
**Yemen**	DALYs	1990-2019	1.30(1.26-1.37)	< 0.001
**Yemen**	DALYs	2019-2023	2.54(2.10-2.85)	< 0.001
**Middle East and North Africa**	Deaths	1990-2019	0.46(0.40-0.57)	< 0.001
**Middle East and North Africa**	Deaths	2019-2023	-0.27(-0.65-0.00)	< 0.001
**Afghanistan**	Deaths	1990-2019	0.98(0.92-1.05)	< 0.001
**Afghanistan**	Deaths	2019-2023	-1.74(-2.16--1.31)	< 0.001
**Algeria**	Deaths	1990-2019	0.61(0.42-0.80)	< 0.001
**Algeria**	Deaths	2019-2023	2.95(1.55-4.29)	< 0.001
**Bahrain**	Deaths	1990-2019	0.59(0.28-0.84)	< 0.001
**Bahrain**	Deaths	2019-2023	0.07(-1.53-2.04)	0.594
**Egypt**	Deaths	1990-2019	2.11(1.81-2.39)	< 0.001
**Egypt**	Deaths	2019-2023	-4.44(-6.40--2.68)	< 0.001
**Iran**	Deaths	1990-2019	1.19(0.77-1.54)	< 0.001
**Iran**	Deaths	2019-2023	0.93(-1.55-3.45)	0.096
**Iraq**	Deaths	1990-2019	0.95(0.81-1.16)	< 0.001
**Iraq**	Deaths	2019-2023	0.11(-1.56-0.94)	0.598
**Jordan**	Deaths	1990-2019	-1.33(-1.55--1.18)	< 0.001
**Jordan**	Deaths	2019-2023	-2.05(-3.20--0.79)	< 0.001
**Kuwait**	Deaths	1990-2019	0.52(-0.14-1.46)	> 0.05
**Kuwait**	Deaths	2019-2023	-2.92(-9.71-3.55)	0.051
**Lebanon**	Deaths	1990-2019	-0.93(-1.36--0.48)	< 0.001
**Lebanon**	Deaths	2019-2023	0.44(-0.33-2.25)	> 0.05
**Libya**	Deaths	1990-2019	0.67(0.54-0.86)	< 0.001
**Libya**	Deaths	2019-2023	-3.33(-4.60--2.43)	< 0.001
**Morocco**	Deaths	1990-2019	1.09(1.01-1.17)	< 0.001

**Morocco**	Deaths	2019-2023	-3.23(-3.80--2.63)	< 0.001
**Oman**	Deaths	1990-2019	0.60(0.39-0.78)	< 0.001
**Oman**	Deaths	2019-2023	0.53(-0.77-2.08)	> 0.05
**Palestine**	Deaths	1990-2019	0.55(0.32-0.81)	< 0.001
**Palestine**	Deaths	2019-2023	1.20(0.84-2.29)	< 0.001
**Qatar**	Deaths	1990-2019	-2.05(-2.53--1.62)	< 0.001
**Qatar**	Deaths	2019-2023	1.99(-2.23-5.15)	> 0.05
**Saudi Arabia**	Deaths	1990-2019	2.42(2.26-2.62)	< 0.001
**Saudi Arabia**	Deaths	2019-2023	3.66(2.12-4.76)	< 0.001
**Sudan**	Deaths	1990-2019	0.82(0.75-0.90)	< 0.001
**Sudan**	Deaths	2019-2023	-0.43(-1.05-0.26)	> 0.05
**Syria**	Deaths	1990-2019	0.24(-0.04-0.47)	> 0.05
**Syria**	Deaths	2019-2023	-2.48(-4.96-0.35)	> 0.05
**Tunisia**	Deaths	1990-2019	-1.11(-1.39--0.65)	< 0.001
**Tunisia**	Deaths	2019-2023	1.57(-1.23-3.90)	> 0.05
**Türkiye**	Deaths	1990-2019	-0.95(-1.12--0.75)	< 0.001
**Türkiye**	Deaths	2019-2023	2.81(1.49-3.52)	< 0.001
**United Arab Emirates**	Deaths	1990-2019	-3.09(-3.38--2.74)	< 0.001
**United Arab Emirates**	Deaths	2019-2023	4.75(2.37-6.71)	< 0.001
**Yemen**	Deaths	1990-2019	0.40(0.28-0.57)	< 0.001
**Yemen**	Deaths	2019-2023	-1.09(-1.91--0.27)	< 0.001
**Middle East and North Africa**	Incidence	1990-2019	1.95(1.93-1.98)	< 0.001
**Middle East and North Africa**	Incidence	2019-2023	2.26(2.11-2.37)	< 0.001
**Afghanistan**	Incidence	1990-2019	1.35(1.34-1.37)	< 0.001
**Afghanistan**	Incidence	2019-2023	1.70(1.60-1.79)	< 0.001
**Algeria**	Incidence	1990-2019	1.73(1.71-1.76)	< 0.001
**Algeria**	Incidence	2019-2023	2.57(2.36-2.75)	< 0.001
**Bahrain**	Incidence	1990-2019	1.59(1.57-1.63)	< 0.001
**Bahrain**	Incidence	2019-2023	1.18(0.96-1.35)	< 0.001
**Egypt**	Incidence	1990-2019	2.62(2.61-2.63)	< 0.001
**Egypt**	Incidence	2019-2023	2.56(2.48-2.63)	< 0.001
**Iran**	Incidence	1990-2019	1.74(1.70-1.79)	< 0.001
**Iran**	Incidence	2019-2023	2.80(2.40-3.12)	< 0.001
**Iraq**	Incidence	1990-2019	1.79(1.78-1.81)	< 0.001
**Iraq**	Incidence	2019-2023	1.91(1.74-2.05)	< 0.001
**Jordan**	Incidence	1990-2019	1.22(1.21-1.24)	< 0.001
**Jordan**	Incidence	2019-2023	0.79(0.75-0.81)	< 0.001
**Kuwait**	Incidence	1990-2019	1.52(1.51-1.54)	< 0.001
**Kuwait**	Incidence	2019-2023	1.85(1.78-1.97)	< 0.001
**Lebanon**	Incidence	1990-2019	1.52(1.50-1.53)	< 0.001
**Lebanon**	Incidence	2019-2023	2.56(2.42-2.64)	< 0.001
**Libya**	Incidence	1990-2019	2.19(2.16-2.22)	< 0.001
**Libya**	Incidence	2019-2023	2.59(2.39-2.81)	< 0.001
**Morocco**	Incidence	1990-2019	1.62(1.61-1.64)	< 0.001
**Morocco**	Incidence	2019-2023	2.25(2.11-2.36)	< 0.001
**Oman**	Incidence	1990-2019	1.69(1.67-1.70)	< 0.001
**Oman**	Incidence	2019-2023	1.83(1.72-2.02)	< 0.001
**Palestine**	Incidence	1990-2019	1.77(1.74-1.80)	< 0.001
**Palestine**	Incidence	2019-2023	0.95(0.72-1.24)	< 0.001
**Qatar**	Incidence	1990-2019	1.55(1.52-1.57)	< 0.001
**Qatar**	Incidence	2019-2023	0.32(0.11-0.49)	< 0.001
**Saudi Arabia**	Incidence	1990-2019	2.08(2.06-2.11)	< 0.001
**Saudi Arabia**	Incidence	2019-2023	2.07(1.82-2.24)	< 0.001
**Sudan**	Incidence	1990-2019	1.69(1.68-1.70)	< 0.001
**Sudan**	Incidence	2019-2023	2.52(2.41-2.63)	< 0.001
**Syria**	Incidence	1990-2019	1.91(1.90-1.92)	< 0.001
**Syria**	Incidence	2019-2023	2.93(2.89-2.99)	< 0.001
**Tunisia**	Incidence	1990-2019	1.23(1.22-1.25)	< 0.001
**Tunisia**	Incidence	2019-2023	1.68(1.55-1.75)	< 0.001
**Türkiye**	Incidence	1990-2019	1.31(1.16-1.41)	< 0.001
**Türkiye**	Incidence	2019-2023	1.37(0.15-1.81)	< 0.001
**United Arab Emirates**	Incidence	1990-2019	1.31(1.28-1.33)	< 0.001
**United Arab Emirates**	Incidence	2019-2023	1.61(1.42-1.79)	< 0.001
**Yemen**	Incidence	1990-2019	2.31(2.29-2.34)	< 0.001
**Yemen**	Incidence	2019-2023	5.50(5.37-5.73)	< 0.001
**Middle East and North Africa**	Prevalence	1990-2019	2.07(2.05-2.09)	< 0.001
**Middle East and North Africa**	Prevalence	2019-2023	2.69(2.51-2.82)	< 0.001
**Afghanistan**	Prevalence	1990-2019	1.58(1.57-1.59)	< 0.001
**Afghanistan**	Prevalence	2019-2023	1.78(1.70-1.86)	< 0.001
**Algeria**	Prevalence	1990-2019	1.81(1.79-1.85)	< 0.001

**Algeria**	Prevalence	2019-2023	2.81(2.56-2.90)	< 0.001
**Bahrain**	Prevalence	1990-2019	1.89(1.87-1.92)	< 0.001
**Bahrain**	Prevalence	2019-2023	1.69(1.50-1.84)	< 0.001
**Egypt**	Prevalence	1990-2019	2.82(2.82-2.83)	< 0.001
**Egypt**	Prevalence	2019-2023	3.00(2.94-3.06)	< 0.001
**Iran**	Prevalence	1990-2019	1.76(1.72-1.80)	< 0.001
**Iran**	Prevalence	2019-2023	2.88(2.49-3.24)	< 0.001
**Iraq**	Prevalence	1990-2019	2.01(1.99-2.02)	< 0.001
**Iraq**	Prevalence	2019-2023	2.04(1.91-2.15)	< 0.001
**Jordan**	Prevalence	1990-2019	1.39(1.36-1.42)	< 0.001
**Jordan**	Prevalence	2019-2023	1.41(1.17-1.57)	< 0.001
**Kuwait**	Prevalence	1990-2019	1.72(1.71-1.75)	< 0.001
**Kuwait**	Prevalence	2019-2023	2.41(2.25-2.63)	< 0.001
**Lebanon**	Prevalence	1990-2019	1.72(1.69-1.75)	< 0.001
**Lebanon**	Prevalence	2019-2023	2.82(2.63-3.05)	< 0.001
**Libya**	Prevalence	1990-2019	2.36(2.32-2.40)	< 0.001
**Libya**	Prevalence	2019-2023	3.05(2.76-3.34)	< 0.001
**Morocco**	Prevalence	1990-2019	1.75(1.74-1.77)	< 0.001
**Morocco**	Prevalence	2019-2023	2.40(2.32-2.47)	< 0.001
**Oman**	Prevalence	1990-2019	1.95(1.93-1.97)	< 0.001
**Oman**	Prevalence	2019-2023	2.19(2.09-2.38)	< 0.001
**Palestine**	Prevalence	1990-2019	2.05(2.02-2.07)	< 0.001
**Palestine**	Prevalence	2019-2023	1.21(1.02-1.51)	< 0.001
**Qatar**	Prevalence	1990-2019	1.69(1.67-1.72)	< 0.001
**Qatar**	Prevalence	2019-2023	0.57(0.38-0.74)	< 0.001
**Saudi Arabia**	Prevalence	1990-2019	2.38(2.34-2.41)	< 0.001
**Saudi Arabia**	Prevalence	2019-2023	2.32(1.99-2.59)	< 0.001
**Sudan**	Prevalence	1990-2019	1.84(1.81-1.88)	< 0.001
**Sudan**	Prevalence	2019-2023	2.68(2.44-2.94)	< 0.001
**Syria**	Prevalence	1990-2019	2.15(2.13-2.16)	< 0.001
**Syria**	Prevalence	2019-2023	3.16(3.07-3.28)	< 0.001
**Tunisia**	Prevalence	1990-2019	1.38(1.37-1.39)	< 0.001
**Tunisia**	Prevalence	2019-2023	1.94(1.89-1.99)	< 0.001
**Türkiye**	Prevalence	1990-2019	1.44(1.25-1.66)	< 0.001
**Türkiye**	Prevalence	2019-2023	2.80(1.31-3.77)	< 0.001
**United Arab Emirates**	Prevalence	1990-2019	1.65(1.63-1.67)	< 0.001
**United Arab Emirates**	Prevalence	2019-2023	2.03(1.87-2.21)	< 0.001
**Yemen**	Prevalence	1990-2019	2.64(2.62-2.67)	< 0.001
**Yemen**	Prevalence	2019-2023	5.89(5.78-6.08)	< 0.001

Table S8. Risk-factor-attributable age-standardized diabetes DALY rates (per 100,000) with 95% uncertainty intervals across 21 Middle East and North Africa countries, 2023.

Country	High BMI	PM pollution	Low phys. activity	Smoking	2nd-hand smoke	Diet: SSBs	Diet: low whole grains	Diet: proc. meat	Diet: red meat	Diet: low fruit	Diet: low veg	Diet: low fiber
**Bahrain**	2208.5 [1084.1–3138.6]	606.1 [327.9–893.7]	379.5 [166.5–593.3]	245.7 [162.6–350.5]	154.8 [82.0–235.9]	203.4 [44.3–466.3]	164.4 [28.1–337.6]	161.0 [55.7–323.7]	253.8 [-44.5–652.6]	49.0 [2.7–161.5]	4.5 [-1.7–11.4]	11.1 [1.2–30.7]
**Saudi Arabia**	1813.6 [951.2–2586.9]	513.8 [326.3–737.9]	292.8 [124.4–444.8]	147.0 [96.2–209.4]	112.0 [58.6–168.9]	293.5 [105.6–529.0]	33.6 [5.4–77.2]	72.3 [29.9–139.5]	48.1 [-6.9–134.4]	72.1 [8.4–173.5]	5.0 [-1.5–12.0]	7.7 [1.0–20.4]
**Kuwait**	1465.2 [784.3–2008.5]	352.2 [208.8–519.8]	291.7 [123.9–451.3]	152.3 [93.1–228.8]	100.5 [52.8–152.8]	136.8 [30.8–291.9]	141.7 [31.0–263.4]	118.3 [44.2–228.0]	116.9 [-16.9–293.3]	37.9 [3.5–108.8]	2.0 [-0.7–5.9]	7.8 [1.7–19.2]
**Iraq**	1499.8 [777.5–2056.5]	438.6 [268.1–632.2]	263.0 [128.6–421.5]	218.9 [153.4–301.1]	137.1 [77.4–201.7]	26.0 [5.1–70.2]	76.7 [13.3–157.6]	74.8 [25.6–147.2]	16.8 [-1.7–50.8]	62.3 [6.7–151.0]	3.3 [-1.1–7.6]	14.3 [3.9–31.4]
**Qatar**	1384.5 [732.3–1909.1]	360.2 [213.7–515.0]	234.3 [106.1–367.8]	113.7 [73.1–173.0]	89.9 [47.1–135.0]	219.3 [62.5–399.9]	104.1 [18.2–208.9]	137.3 [49.2–253.9]	107.2 [-17.6–297.7]	18.6 [0.6–70.2]	1.3 [-0.5–5.0]	2.9 [0.1–10.5]
**Palestine**	1428.0 [719.8–2003.6]	381.2 [236.4–551.1]	217.9 [89.3–336.0]	204.0 [147.7–269.5]	113.4 [61.6–170.4]	9.0 [1.4–25.0]	115.5 [20.0–246.2]	54.3 [20.6–102.6]	27.7 [-3.8–92.6]	71.5 [7.4–183.8]	13.9 [-3.1–91.6]	33.8 [10.6–66.8]
**Egypt**	1459.5 [748.8–2024.2]	406.1 [255.4–576.6]	188.8 [81.3–307.9]	174.2 [122.6–239.6]	108.4 [58.5–161.3]	83.8 [25.4–180.1]	7.8 [1.0–18.3]	75.1 [31.7–145.4]	54.4 [-7.5–143.9]	16.5 [0.9–60.3]	1.1 [-0.3–3.9]	2.3 [0.1–8.4]
**Oman**	1224.9 [632.3–1780.5]	370.6 [228.4–542.8]	200.2 [87.3–315.4]	84.3 [55.6–120.8]	71.1 [35.2–108.3]	43.2 [9.0–119.3]	107.2 [21.6–216.4]	124.2 [43.7–237.0]	100.2 [-11.4–256.0]	7.3 [0.2–33.5]	2.4 [-0.8–6.4]	4.1 [0.5–11.8]
**Jordan**	1071.1 [554.4–1471.0]	299.1 [184.6–430.7]	143.1 [57.7–233.3]	179.9 [125.4–248.5]	95.7 [51.8–141.2]	48.7 [9.7–130.6]	101.1 [21.7–187.1]	33.0 [13.4–61.9]	32.1 [-3.7–89.5]	65.8 [8.7–133.7]	2.4 [-0.8–5.6]	15.4 [5.1–31.4]
**United Arab Emirates**	1050.6 [539.1–1512.9]	302.2 [179.7–443.3]	163.3 [71.2–266.8]	56.3 [32.3–88.7]	66.8 [34.2–101.8]	139.7 [48.9–272.6]	129.4 [30.2–229.5]	64.3 [25.6–121.9]	56.9 [-8.3–145.7]	42.9 [5.0–102.8]	3.0 [-0.9–7.0]	8.0 [1.7–18.8]
**Tunisia**	1135.7 [579.0–1592.8]	292.0 [175.5–431.1]	134.8 [60.8–222.7]	162.4 [113.7–223.3]	106.8 [61.2–157.0]	93.0 [19.8–218.8]	39.5 [6.5–83.4]	35.2 [14.0–63.9]	35.6 [-3.6–104.9]	16.7 [1.0–57.8]	1.2 [-0.4–4.1]	2.6 [0.2–8.0]
**Libya**	1040.0 [538.8–1483.7]	234.5 [135.2–361.9]	146.6 [62.9–243.5]	130.8 [83.7–185.8]	105.6 [60.5–161.5]	35.7 [6.9–98.2]	88.8 [18.2–170.2]	51.8 [20.0–103.8]	29.1 [-3.3–83.2]	16.2 [1.1–52.1]	2.3 [-0.8–5.4]	8.5 [2.1–20.0]
**Syria**	959.4 [484.9–1345.7]	265.4 [162.9–379.8]	140.6 [59.3–231.0]	119.3 [74.5–177.9]	75.5 [40.7–115.4]	30.8 [5.9–83.4]	53.9 [9.7–109.5]	23.2 [9.3–39.9]	33.1 [-3.6–95.2]	27.9 [2.9–76.8]	2.5 [-0.8–6.3]	8.9 [2.5–19.9]
**Lebanon**	882.2 [468.3–1284.3]	232.9 [137.7–345.3]	120.6 [47.4–198.4]	176.2 [121.1–244.5]	78.7 [40.1–119.2]	37.9 [7.4–97.2]	75.1 [15.1–147.1]	34.4 [13.0–69.0]	37.1 [-4.1–107.5]	19.8 [1.7–60.9]	1.4 [-0.5–3.7]	6.0 [1.2–14.4]
**Türkiye**	856.4 [421.9–1191.2]	229.4 [138.9–330.4]	119.0 [47.2–197.8]	119.0 [81.1–162.2]	61.5 [32.5–91.1]	94.2 [29.8–198.1]	16.9 [2.5–40.3]	38.7 [17.3–72.2]	46.1 [-6.8–118.1]	2.9 [0.1–13.3]	0.2 [-0.0–1.2]	1.0 [0.0–3.9]
**Afghanistan**	750.4 [351.7–1152.1]	256.8 [161.9–369.3]	134.8 [58.1–235.2]	42.9 [24.4–70.8]	52.3 [28.6–84.8]	5.4 [0.8–16.0]	34.9 [5.0–77.5]	23.0 [9.2–40.6]	20.3 [-2.6–55.8]	72.7 [9.7–137.5]	80.3 [-27.9–221.0]	16.0 [5.7–33.3]
**Algeria**	834.6 [420.6–1250.7]	196.0 [113.7–298.7]	139.7 [61.1–238.6]	81.7 [50.0–124.4]	79.0 [44.8–120.4]	43.7 [8.4–112.8]	24.3 [3.5–53.2]	33.9 [13.2–66.9]	36.6 [-3.9–103.7]	8.5 [0.4–33.0]	1.7 [-0.6–4.3]	2.3 [0.2–7.0]
**Iran**	716.0 [372.8–1027.2]	207.8 [127.6–306.7]	99.2 [39.2–155.7]	67.7 [40.9–98.1]	37.6 [18.8–55.4]	35.8 [7.2–91.6]	42.2 [7.2–85.7]	32.5 [12.0–62.5]	20.4 [-2.8–57.6]	2.8 [0.1–13.8]	1.4 [-0.5–3.7]	4.7 [0.9–11.3]
**Morocco**	723.0 [380.6–1073.7]	189.0 [114.6–290.2]	120.8 [53.7–188.7]	49.8 [30.3–72.0]	54.9 [29.8–88.9]	31.9 [9.5–71.4]	2.8 [0.3–6.5]	32.7 [13.3–63.6]	36.6 [-4.8–98.6]	10.5 [0.7–38.6]	1.7 [-0.6–4.1]	0.3 [0.0–1.6]
**Sudan**	610.1 [298.7–966.7]	203.5 [118.8–310.7]	189.4 [86.4–318.3]	32.5 [18.1–50.1]	37.7 [20.8–57.7]	10.7 [2.0–31.6]	2.8 [0.4–7.8]	18.7 [7.6–35.0]	40.6 [-6.3–105.4]	41.8 [5.8–84.4]	1.8 [-0.6–4.5]	3.3 [0.4–9.4]
**Yemen**	524.3 [243.9–817.7]	187.3 [114.4–275.2]	100.9 [45.1–169.7]	65.6 [37.5–100.4]	48.4 [26.3–72.8]	3.3 [0.5–9.4]	14.9 [2.1–34.7]	17.0 [6.7–31.3]	10.9 [-1.2–31.4]	72.6 [11.8–132.0]	79.0 [-28.5–207.2]	9.2 [2.8–19.6]

Table S9. Decomposition of diabetes DALYs into years lived with disability (YLDs) and years of life lost (YLLs) by country, 2023.

Country	YLD Age-std Rate* (per 100k) (95% UI)	YLL Age-std Rate* (per 100k) (95% UI)	% YLD (Disability Contribution)
Afghanistan	775.49 (535.85, 1052.16)	597.58 (357.65, 883.68)	56.5%
Algeria	870.69 (603.14, 1170.34)	474.54 (285.16, 718.34)	64.7%
Bahrain	1469.22 (1006.17, 1986.78)	1991.39 (1536.54, 2510.35)	42.5%
Egypt	1146.79 (779.08, 1541.01)	935.65 (718.78, 1214.59)	55.1%
Iran	687.40 (489.02, 939.55)	393.70 (287.43, 508.50)	63.6%
Iraq	1018.78 (694.26, 1407.25)	1242.52 (903.09, 1595.50)	45.1%
Jordan	915.72 (638.42, 1258.54)	641.65 (530.16, 767.31)	58.8%
Kuwait	1419.91 (971.43, 1974.99)	633.16 (562.96, 718.07)	69.2%
Lebanon	1045.59 (725.81, 1399.99)	231.92 (181.51, 290.88)	81.8%
Libya	1056.31 (727.90, 1465.09)	436.97 (260.11, 689.66)	70.7%
Morocco	773.75 (518.65, 1061.37)	407.69 (240.90, 631.00)	65.5%
Oman	855.28 (579.09, 1184.99)	1056.44 (745.74, 1378.35)	44.7%
Palestine	986.52 (683.26, 1345.60)	1118.07 (865.57, 1322.04)	46.9%
Qatar	1175.43 (802.50, 1585.72)	827.00 (654.66, 1044.66)	58.7%
Saudi Arabia	1559.69 (1079.45, 2109.93)	1088.90 (817.79, 1370.90)	58.9%
Sudan	608.98 (407.86, 845.98)	459.01 (281.97, 719.98)	57.0%
Syria	968.42 (671.10, 1352.45)	415.59 (302.21, 572.60)	70.0%
Tunisia	807.89 (550.81, 1130.41)	995.62 (802.29, 1253.06)	44.8%
Türkiye	740.44 (506.25, 983.82)	521.77 (402.52, 652.74)	58.7%
United Arab Emirates	1104.31 (760.84, 1493.60)	477.59 (354.31, 622.98)	69.8%
Yemen	562.24 (381.73, 768.24)	411.45 (261.26, 612.18)	57.7%

Figure S1. Male-to-female ratio of age-specific diabetes DALY rates by age group, 1990 versus 2023.

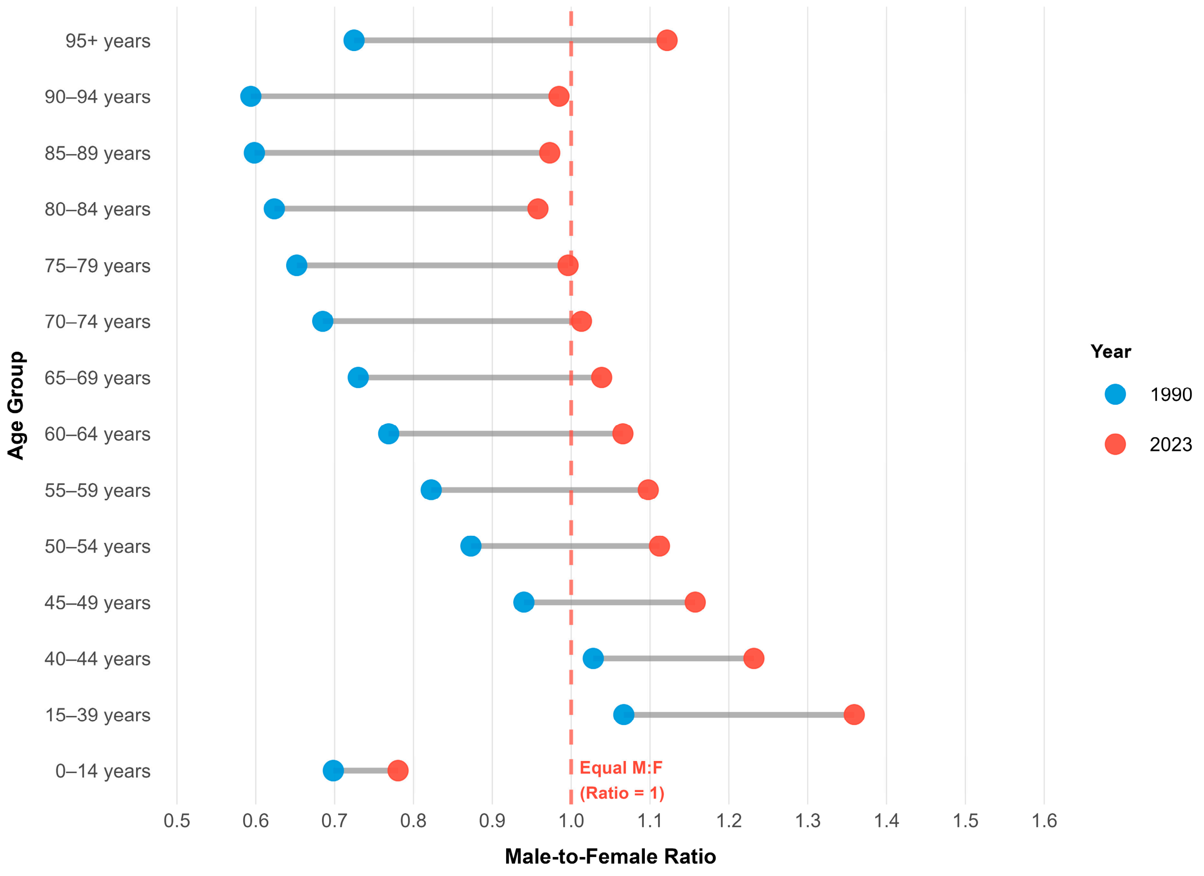

Figure S2. Joinpoint-fitted trends in age-standardized diabetes disability-adjusted life-year (DALY) rates for the remaining 16 Middle East and North Africa countries not shown in Figure 4, 1990–2023.

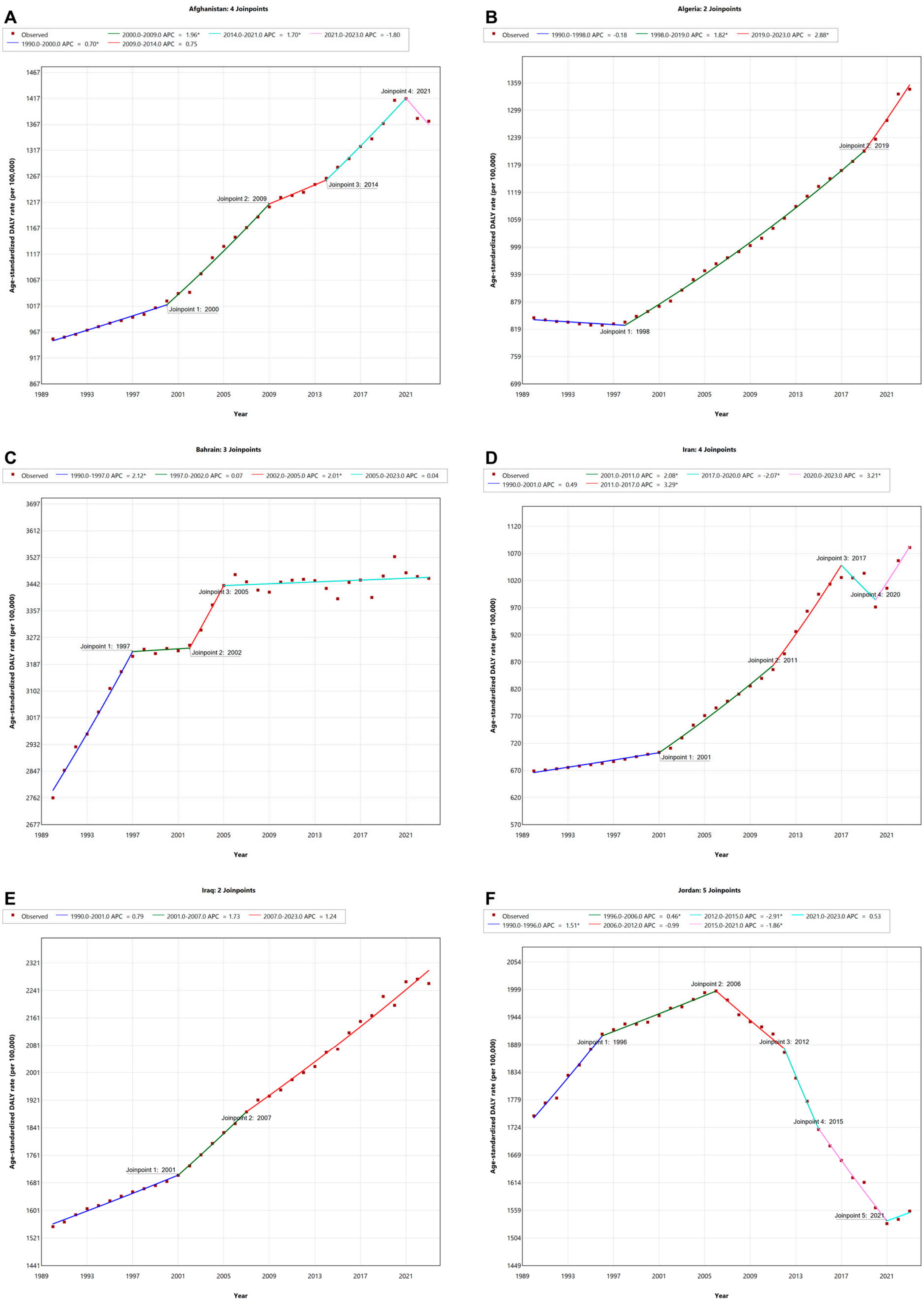

G

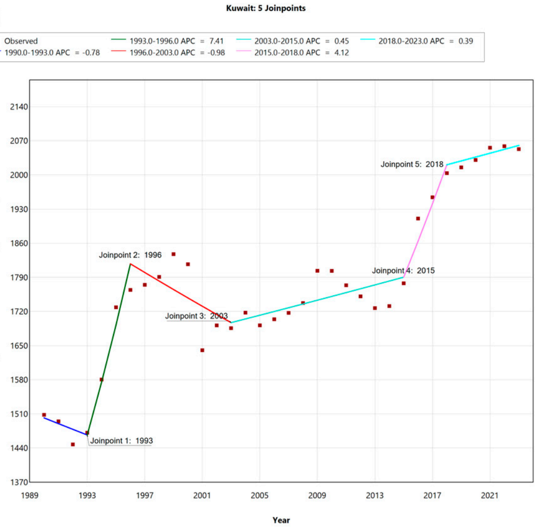
Kuwait: 5 Joinpoints
Observed
1990.0-1993.0 APC = -0.78
1993.0-1996.0 APC = 7.41
1996.0-2003.0 APC = -0.98
2003.0-2015.0 APC = 0.45
2015.0-2018.0 APC = 4.12
2018.0-2023.0 APC = 0.39
Joinpoint 1: 1993
Joinpoint 2: 1996
Joinpoint 3: 2003
Joinpoint 4: 2015
Joinpoint 5: 2018
Age-standardized DALY rate (per 100,000)
Year

H

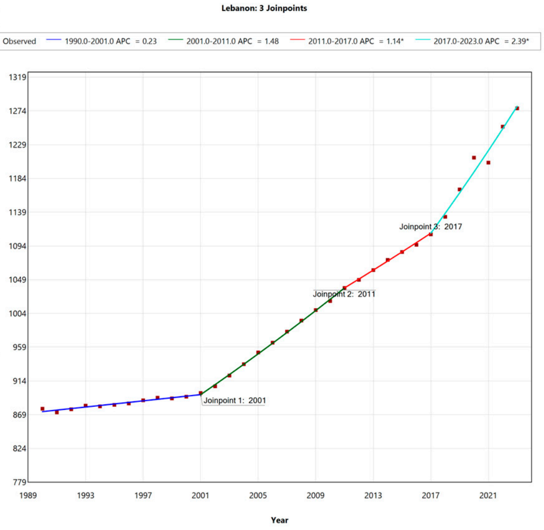
Lebanon: 3 Joinpoints
Observed
1990.0-2001.0 APC = 0.23
2001.0-2011.0 APC = 1.48
2011.0-2017.0 APC = 1.14*
2017.0-2023.0 APC = 2.39*
Joinpoint 1: 2001
Joinpoint 2: 2011
Joinpoint 3: 2017
Age-standardized DALY rate (per 100,000)
Year

I

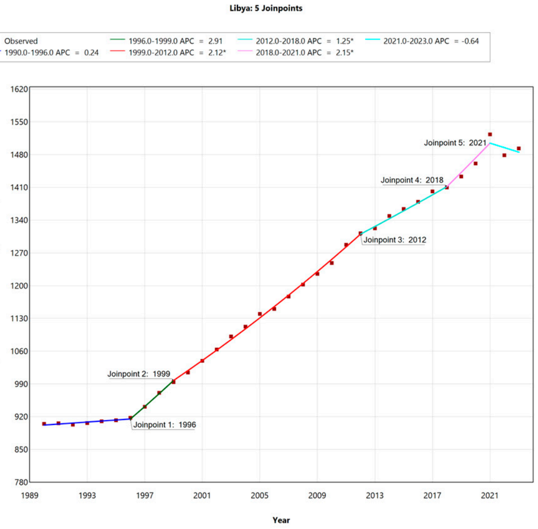
Libya: 5 Joinpoints
Observed
1990.0-1996.0 APC = 0.24
1996.0-1999.0 APC = 2.91
1999.0-2012.0 APC = 2.12*
2012.0-2018.0 APC = 1.25*
2018.0-2021.0 APC = 2.15*
2021.0-2023.0 APC = -0.64
Joinpoint 1: 1996
Joinpoint 2: 1999
Joinpoint 3: 2012
Joinpoint 4: 2018
Joinpoint 5: 2021
Age-standardized DALY rate (per 100,000)
Year

J

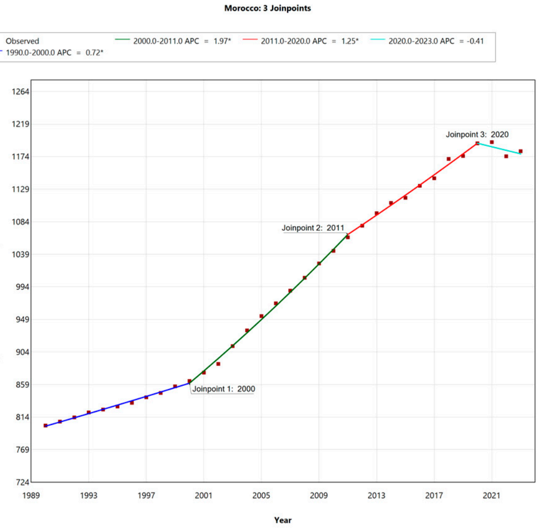
Morocco: 3 Joinpoints
Observed
1990.0-2000.0 APC = 0.72*
2000.0-2011.0 APC = 1.97*
2011.0-2020.0 APC = 1.25*
2020.0-2023.0 APC = -0.41
Joinpoint 1: 2000
Joinpoint 2: 2011
Joinpoint 3: 2020
Age-standardized DALY rate (per 100,000)
Year

K

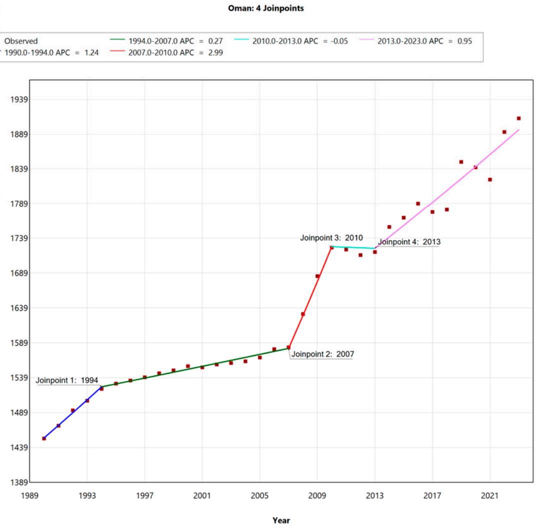
Oman: 4 Joinpoints
Observed
1990.0-1994.0 APC = 1.24
1994.0-2007.0 APC = 0.27
2007.0-2010.0 APC = 2.99
2010.0-2013.0 APC = -0.05
2013.0-2023.0 APC = 0.95
Joinpoint 1: 1994
Joinpoint 2: 2007
Joinpoint 3: 2010
Joinpoint 4: 2013
Age-standardized DALY rate (per 100,000)
Year

L

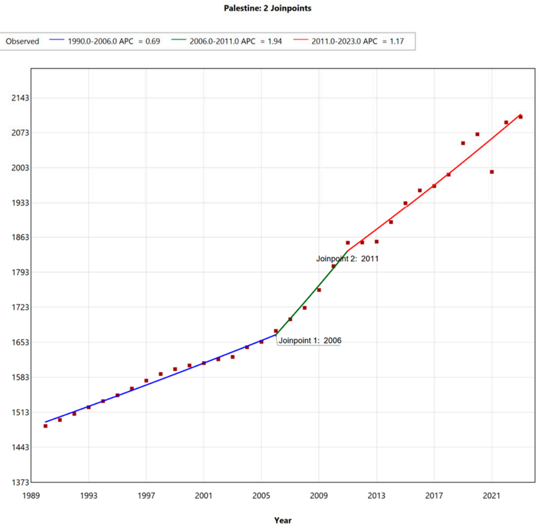
Palestine: 2 Joinpoints
Observed
1990.0-2006.0 APC = 0.69
2006.0-2011.0 APC = 1.94
2011.0-2023.0 APC = 1.17
Joinpoint 1: 2006
Joinpoint 2: 2011
Age-standardized DALY rate (per 100,000)
Year

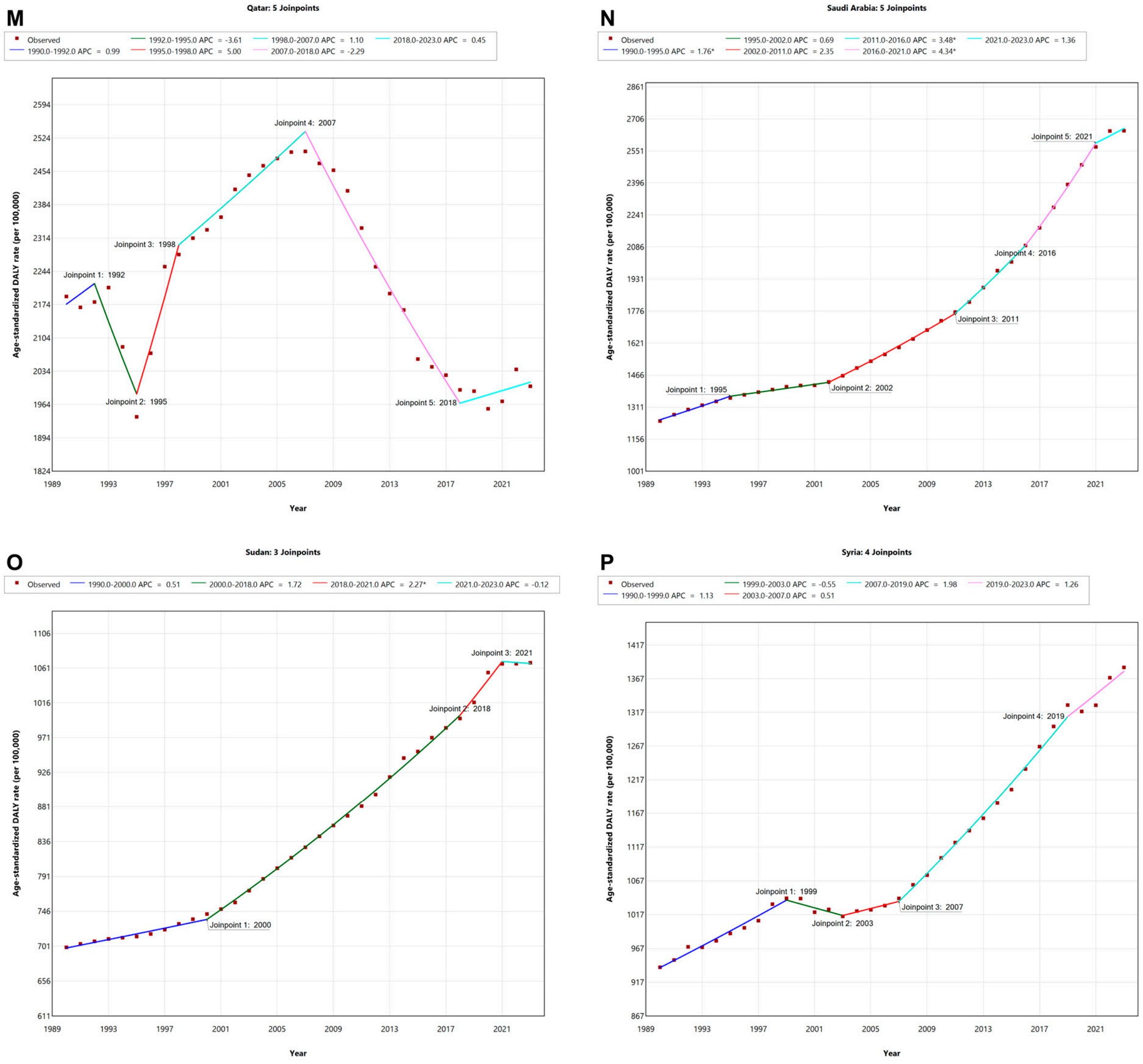
M
Qatar: 5 Joinpoints
Observed
1990.0-1992.0 APC = 0.99
1992.0-1995.0 APC = -3.61
1995.0-1998.0 APC = 5.00
1998.0-2007.0 APC = 1.10
2007.0-2018.0 APC = -2.29
2018.0-2023.0 APC = 0.45
Joinpoint 1: 1992
Joinpoint 2: 1995
Joinpoint 3: 1998
Joinpoint 4: 2007
Joinpoint 5: 2018
Age-standardized DALY rate (per 100,000)
Year
N
Saudi Arabia: 5 Joinpoints
Observed
1990.0-1995.0 APC = 1.76*
1995.0-2002.0 APC = 0.69
2002.0-2011.0 APC = 2.35
2011.0-2016.0 APC = 3.48*
2016.0-2021.0 APC = 4.34*
2021.0-2023.0 APC = 1.36
Joinpoint 1: 1995
Joinpoint 2: 2002
Joinpoint 3: 2011
Joinpoint 4: 2016
Joinpoint 5: 2021
Age-standardized DALY rate (per 100,000)
Year
O
Sudan: 3 Joinpoints
Observed
1990.0-2000.0 APC = 0.51
2000.0-2018.0 APC = 1.72
2018.0-2021.0 APC = 2.27*
2021.0-2023.0 APC = -0.12
Joinpoint 1: 2000
Joinpoint 2: 2018
Joinpoint 3: 2021
Age-standardized DALY rate (per 100,000)
Year
P
Syria: 4 Joinpoints
Observed
1990.0-1999.0 APC = 1.13
1999.0-2003.0 APC = -0.55
2003.0-2007.0 APC = 0.51
2007.0-2019.0 APC = 1.98
2019.0-2023.0 APC = 1.26
Joinpoint 1: 1999
Joinpoint 2: 2003
Joinpoint 3: 2007
Joinpoint 4: 2019
Age-standardized DALY rate (per 100,000)
Year

Figure S3. Country-specific average annual percent changes in age-standardized diabetes burden, MENA region, 1990–2023. Heatmap displaying AAPCs (% per year) for 21 MENA countries across four burden metrics (incidence, prevalence, mortality, DALYs) for the full period (1990–2023). Color intensity represents AAPC magnitude: red shading indicates higher rates of increase; blue shading indicates lower rates of increase or decline. Countries are ordered from lower overall mean AAPC at the top to higher overall mean AAPC at the bottom. AAPC, average annual percent change; DALY, disability-adjusted life-year.

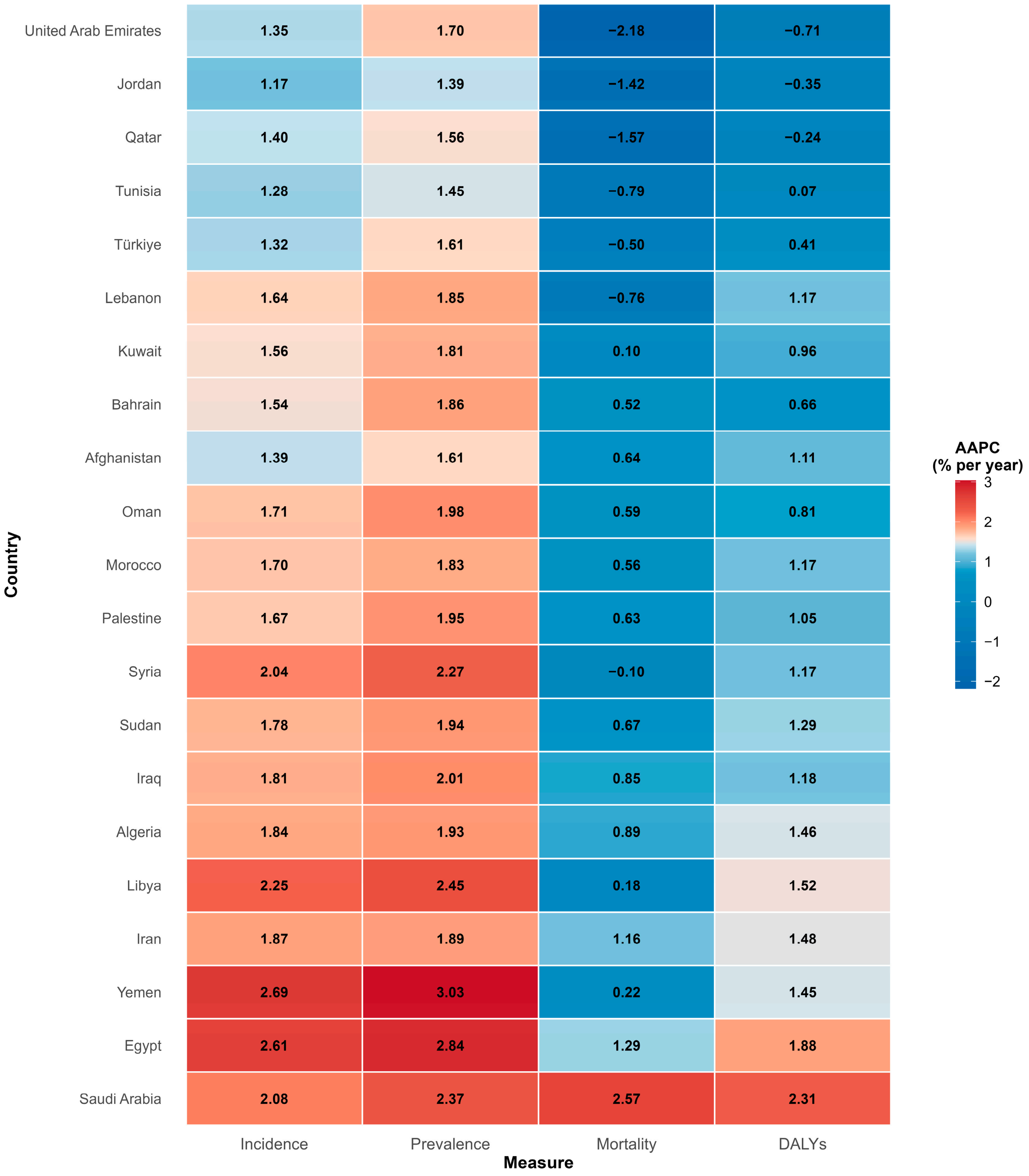

Checklist S1. GATHER checklist.

Item #	Checklist item	Reported on page #
**Objectives and funding**		
1	Define the indicator(s), populations (including age, sex, and geographic entities), and time period(s) for which estimates were made.	Title page, pp. 1-2; Abstract, p. 3; Materials and methods, pp. 5-7.
2	List the funding sources for the work.	This work was supported by JSPS KAKENHI Grant Number 26K02613.
**Data Inputs**		
For all data inputs from multiple sources that are synthesized as part of the study:		
3	Describe how the data were identified and how the data were accessed.	Materials and methods: Study design and data source, p. 5; Data availability, p. 7.
4	Specify the inclusion and exclusion criteria. Identify all ad-hoc exclusions.	Materials and methods: Study design and data source and Case definition, p. 5.
5	Provide information on all included data sources and their main characteristics. For each data source used, report reference information or contact name/institution, population represented, data collection method, year(s) of data collection, sex and age range, diagnostic criteria or measurement method, and sample size, as relevant.	Materials and methods: Study design and data source, Case definition, Epidemiological metrics, Risk factor assessment, and Estimation methods, pp. 5-7.
6	Identify and describe any categories of input data that have potentially important biases (e.g., based on characteristics listed in item 5).	Materials and methods: Estimation methods and Statistical analysis, pp. 6-7; Strengths and limitations, p. 15.
For data inputs that contribute to the analysis but were not synthesized as part of the study:		
7	Describe and give sources for any other data inputs.	Materials and methods: Risk factor assessment and Estimation methods, pp. 6-7.
For all data inputs:		
8	Provide all data inputs in a file format from which data can be efficiently extracted (e.g., a spreadsheet rather than a PDF), including all relevant meta-data listed in item 5. For any data inputs that cannot be shared because of ethical or legal reasons, such as third-party ownership, provide a contact name or the name of the institution that retains the right to the data.	Data availability, p. 7; S1-S7 Tables, p. 24; source data available through the GBD Results Tool.
**Data analysis**		
9	Provide a conceptual overview of the data analysis method. A diagram may be helpful.	Materials and methods: Study design and data source, Risk factor assessment, Estimation methods, and Statistical analysis, pp. 5-7.

**10**	Provide a detailed description of all steps of the analysis, including mathematical formulae. This description should cover, as relevant, data cleaning, data pre-processing, data adjustments and weighting of data sources, and mathematical or statistical model(s).	Materials and methods: Epidemiological metrics, Risk factor assessment, Estimation methods, and Statistical analysis, pp. 6-7.
**11**	Describe how candidate models were evaluated and how the final model(s) were selected.	Materials and methods: Estimation methods and Statistical analysis, pp. 6-7.
**12**	Provide the results of an evaluation of model performance, if done, as well as the results of any relevant sensitivity analysis.	N/A
**13**	Describe methods for calculating uncertainty of the estimates. State which sources of uncertainty were, and were not, accounted for in the uncertainty analysis.	Materials and methods: Epidemiological metrics and Statistical analysis, pp. 6-7; Strengths and limitations, p. 15.
**14**	State how analytic or statistical source code used to generate estimates can be accessed.	Analysis and figure-generation code are deposited at Zenodo (doi: 10.5281/zenodo.19813641).
**Results and Discussion**		
**15**	Provide published estimates in a file format from which data can be efficiently extracted.	Table 1, p. 10; S1-S7 Tables, p. 24; supplementary materials file.
**16**	Report a quantitative measure of the uncertainty of the estimates (e.g. uncertainty intervals).	Abstract, p. 3; Materials and methods: Epidemiological metrics, p. 6; Results, pp. 8-11; Table 1, p. 10; S1-S7 Tables, p. 24.
**17**	Interpret results in light of existing evidence. If updating a previous set of estimates, describe the reasons for changes in estimates.	Introduction, pp. 4-5; Discussion, pp. 11-15.
**18**	Discuss limitations of the estimates. Include a discussion of any modelling assumptions or data limitations that affect interpretation of the estimates.	Strengths and limitations, p. 15.

Checklist S2. RECORD-STROBE checklist for observational studies using routinely collected health data.

	Item No.	STROBE items	Location in manuscript where items are reported	RECORD items	Location in manuscript where items are reported
**Title and abstract**					
	1	(a) Indicate the study's design with a commonly used term in the title or the abstract (b) Provide in the abstract an informative and balanced summary of what was done and what was found	Title page, pp. 1-2; Abstract, p. 3.	RECORD 1.1: The type of data used should be specified in the title or abstract. When possible, the name of the databases used should be included.<br><br>RECORD 1.2: If applicable, the geographic region and timeframe within which the study took place should be reported in the title or abstract.<br><br>RECORD 1.3: If linkage between databases was conducted for the study, this should be clearly stated in the title or abstract.	Title page, pp. 1-2; Abstract, p. 3;
**Introduction**					
Background rationale	2	Explain the scientific background and rationale for the investigation being reported	Introduction, pp. 4-5.		
Objectives	3	State specific objectives, including any prespecified hypotheses	Introduction, p. 5.		
**Methods**					
Study Design	4	Present key elements of study design early in the paper	Materials and methods: Study design and data source, p. 5.		
Setting	5	Describe the setting, locations, and relevant dates, including periods of recruitment, exposure, follow-up, and data collection	Abstract, p. 3; Materials and methods: Study		
Participants	6	*(a) Cohort study* - Give the eligibility criteria, and the sources and methods of selection of participants. Describe methods of follow-up<br><br>*Case-control study* - Give the eligibility criteria, and the sources and methods of case ascertainment and control	N/A<br><br>Materials and methods, pp. 5-6.	RECORD 6.1: The methods of study population selection (such as codes or algorithms used to identify subjects) should be listed in detail. If this is not possible, an explanation should be provided.	Materials and methods: Study design and data source and Case definition, p. 5

		selection. Give the rationale for the choice of cases and controls<br><br>*Cross-sectional study* - Give the eligibility criteria, and the sources and methods of selection of participants<br><br>*(b) Cohort study* - For matched studies, give matching criteria and number of exposed and unexposed<br><br>*Case-control study* - For matched studies, give matching criteria and the number of controls per case		RECORD 6.2: Any validation studies of the codes or algorithms used to select the population should be referenced. If validation was conducted for this study and not published elsewhere, detailed methods and results should be provided.<br><br>RECORD 6.3: If the study involved linkage of databases, consider use of a flow diagram or other graphical display to demonstrate the data linkage process, including the number of individuals with linked data at each stage.	
Variables	7	Clearly define all outcomes, exposures, predictors, potential confounders, and effect modifiers. Give diagnostic criteria, if applicable.	Materials and methods: Case definition, Epidemiological metrics, Risk factor assessment, and Statistical analysis, pp. 5-7.	RECORD 7.1: A complete list of codes and algorithms used to classify exposures, outcomes, confounders, and effect modifiers should be provided. If these cannot be reported, an explanation should be provided.	Materials and methods: Case definition, p. 5; ICD-10 codes E10-E14 are specified.
Data sources/ measurement	8	For each variable of interest, give sources of data and details of methods of assessment (measurement).<br><br>Describe comparability of assessment methods if there is more than one group	Materials and methods: Study design and data source, Epidemiological metrics, Risk factor assessment, and Estimation methods, pp. 5-7; Data availability, p. 7.		
Bias	9	Describe any efforts to address potential sources of bias	Materials and methods: Estimation methods and Statistical analysis, pp. 6-7; Strengths and limitations, p. 15.		
Study size	10	Explain how the study size was arrived at	Materials and methods: Study design and data source, p. 5.		
Quantitative variables	11	Explain how quantitative variables were handled in the analyses. If applicable, describe which groupings were chosen, and why	Materials and methods: Epidemiological metrics and		

			Statistical analysis, pp. 6-7.		
Statistical methods	12	(a) Describe all statistical methods, including those used to control for confounding<br><br>(b) Describe any methods used to examine subgroups and interactions<br><br>(c) Explain how missing data were addressed<br><br>(d) *Cohort study* - If applicable, explain how loss to follow-up was addressed<br><br>*Case-control study* - If applicable, explain how matching of cases and controls was addressed<br><br>*Cross-sectional study* - If applicable, describe analytical methods taking account of sampling strategy<br><br>(e) Describe any sensitivity analyses	Materials and methods: Statistical analysis, p. 7; Results, pp. 8-11.		
Data access and cleaning methods		..	N/A	RECORD 12.1: Authors should describe the extent to which the investigators had access to the database population used to create the study population.<br><br>RECORD 12.2: Authors should provide information on the data cleaning methods used in the study.	Data availability, p. 7; Materials and methods: Statistical analysis, p. 7; Author Contributions, p. 16.
Linkage		..	N/A	RECORD 12.3: State whether the study included person-level, institutional-level, or other data linkage across two or more databases. The methods of linkage and methods of linkage quality evaluation should be provided.	N/A
**Results**					
Participants	13	(a) Report the numbers of individuals at each stage of the study (*e.g.*, numbers potentially eligible, examined for eligibility, confirmed eligible, included in the study, completing follow-up, and analysed)	N/A; Materials and methods, p. 5.	RECORD 13.1: Describe in detail the selection of the persons included in the study (*i.e.,* study population selection) including filtering based on data quality, data availability and linkage. The selection of included persons can be described in the text and/or by means of the study flow diagram.	Materials and methods: Study design and data source, p. 5

		(b) Give reasons for non-participation at each stage.<br><br>(c) Consider use of a flow diagram			
Descriptive data	14	(a) Give characteristics of study participants (*e.g.*, demographic, clinical, social) and information on exposures and potential confounders<br><br>(b) Indicate the number of participants with missing data for each variable of interest<br><br>(c) *Cohort study* - summarise follow-up time (*e.g.*, average and total amount)	Results, pp. 8-11; Table 1, p. 10; S1-S7 Tables, p. 24.		
Outcome data	15	*Cohort study* - Report numbers of outcome events or summary measures over time<br><br>*Case-control study* - Report numbers in each exposure category, or summary measures of exposure<br><br>*Cross-sectional study* - Report numbers of outcome events or summary measures	Results, pp. 8-11; Table 1, p. 10; S1-S4 and S7 Tables, p. 24.		
Main results	16	(a) Give unadjusted estimates and, if applicable, confounder-adjusted estimates and their precision (e.g., 95% confidence interval). Make clear which confounders were adjusted for and why they were included<br><br>(b) Report category boundaries when continuous variables were categorized<br><br>(c) If relevant, consider translating estimates of relative risk into absolute risk for a meaningful time period	Abstract, p. 3; Results, pp. 8-11; Table 1, p. 10.		
Other analyses	17	Report other analyses done—e.g., analyses of subgroups and interactions, and sensitivity analyses	Results, pp. 9-11; Figs 2-4, pp. 9-11; S1 Fig, p. 25; S5-S6 Tables, p. 24.		
**Discussion**					

Key results	18	Summarise key results with reference to study objectives	Discussion, pp. 11-15; Conclusions, p. 16.		
Limitations	19	Discuss limitations of the study, taking into account sources of potential bias or imprecision. Discuss both direction and magnitude of any potential bias	Strengths and limitations, p. 15.	RECORD 19.1: Discuss the implications of using data that were not created or collected to answer the specific research question(s). Include discussion of misclassification bias, unmeasured confounding, missing data, and changing eligibility over time, as they pertain to the study being reported.	Strengths and limitations, p. 15.
Interpretation	20	Give a cautious overall interpretation of results considering objectives, limitations, multiplicity of analyses, results from similar studies, and other relevant evidence	Discussion, pp. 11-15; Conclusions, p. 16.		
Generalisability	21	Discuss the generalisability (external validity) of the study results	Country-level variation and risk factors, p. 14; Strengths and limitations, p. 15.		
**Other Information**					
Funding	22	Give the source of funding and the role of the funders for the present study and, if applicable, for the original study on which the present article is based	Funding statement (after the Conclusions): "This research was funded by JSPS KAKENHI, grant number 26K02613."		
Accessibility of protocol, raw data, and programming code			Data availability, p. 7; S1-S7 Tables, p. 24;	RECORD 22.1: Authors should provide information on how to access any supplemental information such as the study protocol, raw data, or programming code.	Data availability, p. 7; S1-S7 Tables, p. 24;
